# A joint analysis of accessibility and household trip frequencies by travel mode

**DOI:** 10.1016/j.tra.2024.104007

**Published:** 2024-02-23

**Authors:** Abhilash C. Singh, Ahmadreza Faghih Imani, Aruna Sivakumar, Yang Luna Xi, Eric J. Miller

**Affiliations:** a Urban Systems Lab and Centre for Transport Studies, Imperial College London, London SW7 2AZ, United Kingdom; b Centre for Environmental Policy, Imperial College London, London SW7 2AZ, United Kingdom; c University of Toronto Transportation Research Institute, University of Toronto, Toronto M5S 1A4, Canada; d Department of Civil & Mineral Engineering, University of Toronto Transportation Research Institute, University of Toronto, Toronto M5S 1A4, Canada

**Keywords:** Simultaneity, Accessibility, Trip frequencies, Joint econometric model, Endogeneity

## Abstract

This paper examines the endogenous relationship between residential level of accessibility and household trip frequencies to tease out the direct and indirect effects of observed behavioural differences. We estimate a multivariate ordered probit model system, which allows dependence in both observed and unobserved factors, using data from the 2016 Transportation Tomorrow Survey (TTS), a household travel survey in the Greater Golden Horseshoe Area (GGH) in Toronto. The modelling framework is used to analyse the influence of exogenous variables on eight outcome variables of accessibility levels and trip frequencies by four modes (auto, transit, bicycle and walk), and to explore the nature of the relationships between them. The results confirm our hypothesis that not only does a strong correlation exist between the residential level of accessibility and household trip frequency, but there are also direct effects to be observed. The complementarity effect between auto accessibility and transit trips, and the substitution effect observed between transit accessibility and auto trips highlight the residential neighbourhood dissonance of transit riders. It shows that locations with better transit service are not necessarily locations where people who make more transit trips reside. Essentially, both jointness (due to error correlations) as well as directional effects observed between accessibility and trip frequencies of multiple modes offer strong support for the notion that accessibility and trip frequency by mode constitute a bundled choice and need to be considered as such.

## 1 Introduction

### 1.1 Background and motivation

Travel behaviour exhibits a complex relationship with built-environment attributes, which has been extensively studied in the literature (Cervero and Kockelman, 1997; Frank and Engelke, 2001; Bagley and Mokhtarian, 2002; Cervero and Duncan, 2003; Krizek, 2003; Shay and Khattak, 2005; Lee and Moudon, 2006; Copperman and Bhat, 2007; Vance and Hedel, 2007; Brownstone and Golob, 2009; Cao et al., 2009; Cao, 2010; Zhang et al., 2012; He and Zhang, 2014; Liao et al., 2015; Næss et al., 2018). *Neighbourhood-level* accessibility, measuring the impact of the immediate built environment on travel behaviour, may be homogenous across different travel modes, but the influence becomes disproportionate beyond the immediate vicinity. This can be observed through variations in accessibility levels across different travel modes. Earlier studies have focused on immediate neighbourhood characteristics such as the availability of facilities, land use density, and street network features (Handy et al., 2005; Mokhtarian and Cao, 2008; Cao et al., 2009; Singh et al., 2018). However, there are relatively fewer studies that assess the impact of built environment beyond the immediate neighbourhood (Krizek, 2003; Ewing and Cervero, 2010). In fact, one can argue that focusing on network-level accessibility can lead to a wider understanding of the factors influencing travel behaviour, such as city-level transport infrastructure, density and population distribution, network connectivity, distance to destinations, topography, safety, and walkability.

*Network-level accessibility* is defined as the capacity to reach valued destinations where opportunities, activities, and individuals are located. For this research we employ employment or employment opportunities as a proxy for a valued destination. Our use of the term aligns with previous research (Giuliano et al., 2012) which states that *network accessibility* refers to the ability of a location within a network to connect with other locations, with its significance stemming from its impact on the spatial distribution of resources (such as workers and consumers) and generating spatial interaction via transport services. It is typically quantified based on travel impedance and the number of reachable opportunities (Xi et al., 2018). Ewing and Cervero (2010) observed that accessibility to destinations strongly affects household travel behaviour. Accessibility to employment opportunities within an isochrone is the most common measure of accessibility used in regional planning (Boisjoly and El-Geneidy, 2017) and can be found in the analysis of land value (Du and Mulley, 2012; Iacono et al., 2008), commuting distance (Levinson, 1998; Manaugh et al., 2010), social equity (Manaugh and El-Geneidy, 2012; Foth et al., 2013) and mode share (Foth et al., 2014; Owen and Levinson, 2015).

Therefore, we hypothesise that the observed levels of network-level accessibility (represented by a cumulative opportunities approach measured by the number of employments reachable within the isochrones) by different travel modes have a substantial impact on household travel behaviour. This study aims to investigate the direct and indirect effects between travel behaviour and the level of accessibility (represented by level of access to employment opportunities) by empirically analysing the direction and extent of their mutual influence, focusing on multi-modal household trip frequencies and accessibility. In the rest of this paper, we will use the term accessibility, instead of referring to the multi-faceted network-level accessibility, for ease in presentation.

### 1.2 Simultaneity bias in accessibility and travel behaviour

Past research has explored unidirectional effects of the built environment on travel behaviour (De Vos et al., 2019; Haque et al., 2019; Zarabi et al., 2019; Farinloye et al., 2019; Kamruzzaman et al., 2020). However, immediate or delayed feedback from endogenous choices in travel-behaviour analysis requires further inspection. Evidence supporting the prevalence of this type of simultaneity bias has been documented (Guevara and Ben-Akiva, 2006), for instance leading to inconsistent estimation of price coefficients in housing price models (Waddell, 1992; Bhat and Guo, 2004). Simultaneity bias has been observed in various activity travel behaviour models, dating back to the early 1990s (Kitamura et al., 1992; Golob et al., 1994), underlining the importance of considering the simultaneity of choices to avoid erroneous parameter estimates (Pinjari et al., 2011).

According to De Witte et al. (2013), variables used to explain mode choice include the number of household cars, variables linked to the built environment, land use and network accessibility, and travel cost; which is linked to the decision to own a transit pass. Usually, these variables are considered to be explanatory variables for mode choice, which makes it difficult to distinguish between a causal relationship and pure correlation. While one may hypothesise that household car ownership has a significant impact on mode choice, it is also possible that a household has several cars because all adults in the household commute by car. In the same manner, the characteristics of the available transport modes may be strongly linked to the residential location. For instance, some commuters may have poor public transport options available because they live in a low-density zone. Car dependency may then be partly due to a residential self-selection effect. Pinjari et al. (2011) summarise the various interdependencies between this continuum of choices into four categories. First, long-term choices (e.g., residential location) have a direct effect on short-term (mode) choices. Second, the residential self-selection effect is due to the selection of individuals in residential areas based on lifestyle preferences, which are also related to car or transit-pass ownership or to mode choice. Third, bicycle, car or transit-pass ownership is endogenous with respect to mode choice (such as individuals commuting by train buy an annual transit-pass). Fourth, some choices are associative (and not causal) and can be explained by common unobserved latent variables (that is a household whose member has a transit-pass will have fewer cars available, maybe because of positive perceptions towards public transport modes or environmental concerns or proximity to transit). In statistical terms, ignoring the interdependence of the transport related choices, and estimating separate models when there is clear endogeneity, is misleading and may result in inefficient coefficients.

Another important consideration to model development is future forecasts and policy design based on the relationship between residential location and travel behaviour. Forecasts for policy design may be seriously misinformed if the model estimation is inconsistent. An inconsistent estimation approach may invalidate model analysis and has been noted to be unavoidable in models of residential location choice because of endogeneity, unless it is addressed methodically (Guevara and Ben-Akiva, 2006). Given the substantial simultaneity-endogeneity between accessibility and travel behaviour attributes, it is fitting to develop models which could jointly model these as a bundle of choices made simultaneously rather than sequentially.

### 1.3 Contributions of this paper

This paper presents a modelling framework that simultaneously models the level of accessibility and travel behaviour, by different transport modes. Specifically, we generate accessibility measures in the form of cumulative employment opportunities (that is, the total number of employment opportunities that can be reached from the household location) within an isochrone, for each of the four modes of travel: auto, transit, bicycle, and walk. Travel behaviour is then examined by investigating the total number of trips made by the household by each of the four modes of travel. While factors such as price and dwelling attributes may have a greater impact on choice of residential location, the residential location determines the impact of accessibility on travel choices, including number of trips by private vs. public transport, proportion of active transport trips, and total vehicle miles travelled. Employment accessibility has a significant impact on the socio-economic well-being of the households. While other accessibility measures such as walkability index, transit connectivity and land use diversity have been analysed in the literature (Cao and Fan, 2012; Kim and Brownstone, 2013; Paleti et al., 2013; Bhat et al., 2016), modelling employment accessibility and its impacts provides a critical understanding of the socio-economic welfare of a household. We hypothesise that using employment accessibility alone as the endogenous variable may allow us a clean identification of its direct effect on travel behaviour, as well as any remaining self-selection effects, in explaining household trips without problems of multi-collinearity with other accessibility characteristics.

This paper uses a multivariate ordered-response model system for analysing the level of accessibility to employments and the number of trips (trip frequencies) undertaken by a household, respectively, by each mode (auto, transit, bicycle and walk). Both household trip frequencies and accessibility measures are categorised in ordered levels of very low, low, medium, high, and very high. For an analysis like this, household size plays a critical role, as we can expect larger households to prefer suburban neighbourhoods and younger/smaller households to self-select city-centre neighbourhoods. This effect of household size can be accounted for by creating a complex size variable comprising of number of people in the household, age, and gender. However, to explicitly tease out the effect of household size and type in our analysis, we choose to deconstruct the household size effect by comprehensive structuring of household types, including single adult with no children, adult couple with no children, single or couple with one or more children, multi-adult with no children, and multi-adult with one or more children. In this model system, we allow dependence between the trip frequency and accessibility by different modes due to both observed and unobserved factors. The observed factors are accounted for by the exogenous variables available in the dataset. The inclusion of unobserved factors allows complementarity and substitution effects in trip counts (even after controlling for observed effects). For instance, individuals in the higher income bracket may have a higher propensity to driving but still live in transit-rich neighbourhoods. This would create a complementary relationship between frequency of driving trips and transit trips. Similarly, individuals with a more active lifestyle may have a higher propensity to bicycle or walk, but not drive. This represents a substitution relationship. Overall, the extent of complementary and substitution relationships may be specific to the combinations of level of accessibility and trip frequencies, respectively, by each mode.

In this study, we demonstrate the applicability of the modelling framework using travel survey data from the 2016 Transportation Tomorrow Survey (TTS), a household travel survey in the Greater Golden Horseshoe Area (GGH) in Toronto (Data Management Group, 2016). The dataset is augmented with accessibility generated measures by different travel modes for the households’ residential locations. The multivariate ordered probit model is structured in a way to tease out relationships among the endogenous variables (such as accessibility to opportunities by auto mode and auto trip frequency) while explicitly accounting for the influence of exogenous variables such as socio-demographic characteristics and other accessibility attributes. The previously mentioned literature clearly illustrates the need for methodical approaches to disentangle endogeneity. We therefore attempt to disentangle the bidirectional relationship between accessibility and trip frequency in an empirical context. It is crucial to address this gap via a model that simultaneously assesses accessibility and travel behaviour across multiple transportation modes. It allows for a comprehensive understanding of how accessibility to employment opportunities affects household travel behaviour and offers the ability to quantitatively measure self-selection effects between employment accessibility (associated with the household’s specific residential location) and trip frequency, essential for urban planning and transportation policy. The remainder of this paper is organised as follows. Section 2 provides a description of the data. Section 3 presents the model formulation and conceptual framework while Section 4 offers details on model estimation results and goodness-of-fit analysis. Concluding remarks and a discussion of the implications of the findings are presented in Section 5.

## 2 Data description

The data used for this study is a sample taken from the 2016 Transportation Tomorrow Survey (TTS). The TTS is a household travel survey collecting travel information from 5 % of households in the GGH that has been conducted every five years since 1986 (Data Management Group, 2016). The TTS collects individual and household information in addition to data regarding the trips made in a day, such as travel mode, start time, etc. After removing the households with incomplete and inconsistent attributes, the remaining 42,718 households residing in the City of Toronto were analysed. These households made 196,221 trips which are the basis of this analysis. The dependent variables of interest in this paper are the household trip frequency by each mode and the respective modal accessibility measure calculated using home location. Trip records provided by the households were used to derive the total modal trip frequency estimates for each household. The modes chosen by the household were clustered into categories for auto, transit, bicycle and walk; aggregated at the household level. Modal accessibility measures were calculated for each dissemination area (DA) in the City of Toronto and assigned to the households based on the DA of their home location. Dissemination areas are the smallest of Canadian census agglomerations, with populations between 400 and 700 people. We generated travel time isochrones by each mode and calculated the cumulative employment opportunities captured within the isochrone. Different time cut-offs were tested and as a result, 45-minutes was used for auto and transit modes and 30-minutes was used for bicycle and walk modes. Auto and Transit travel times were obtained from GTAModel V4.1 for the morning peak period (Travel Modelling Group, 2015), which accounts for road congestion and actual speeds (rather than posted speed limits). For bicycle and walk isochrones, a uniform speed of 15 km/h and 4 km/h were assumed. For bicycle accessibility measures, we also accounted for Level of Traffic Stress (LTS) on the road network. The LTS metric classifies street segments between LTS 1 (low stress) and LTS 4 (high stress), considering primary roadway attributes including: speed limit, number of vehicular lanes, bicycle lanes, parking, and traffic signals (Furth et al., 2016). We calculated the level of cycling stress for every link in Toronto’s Road network. Using this street stress network, the 30-minute cycling isochrone and associated cumulative opportunities to employments were generated using only the road links with lower stress levels of 1 and 2 (for detailed discussion on cycle accessibility and level of traffic stress, see Faghih Imani et al., 2019). To accommodate household size effect, the dataset comprising of 42,718 households was divided by comprehensive structuring of household types: single adult with no children (13,613 households), adult couple with no children (14,781 households), single or couple with one or more children (6,526 households), multi-adult with no children (5,660 households), and multi-adult with one or more children (2,138 households).

Variables extracted from the TTS survey included trip distance, and household characteristics such as number of vehicles in the household, and household income. In addition to these, proximity to the central business district (CBD) was computed as the distance from the household home location to the Toronto City Hall. Population density and employment density at home location were generated at DA level and appended to the dataset. Table 1 provides an overview of the descriptive characteristics of the sample. Table 2 provides a distribution of the dependent variables as per the ordered scales of very low, low, medium, high, and very high trip frequency.

**Fig. 1:**
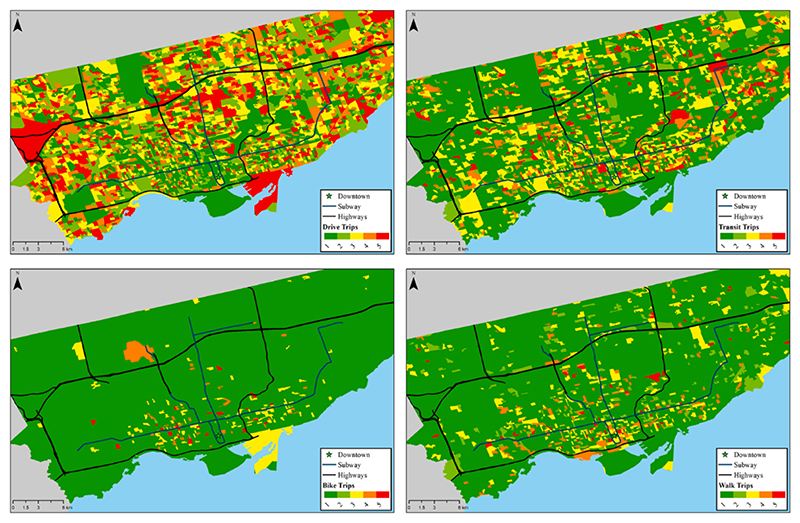
Map of Greater Toronto region with modal accessibilities, clockwise (from top right) the levels of transit, walk, bicycle and auto accessibility (green for lowest category 1, up to red for highest category 5). (For interpretation of the references to colour in this figure legend, the reader is referred to the web version of this article.)

**Fig. 2:**
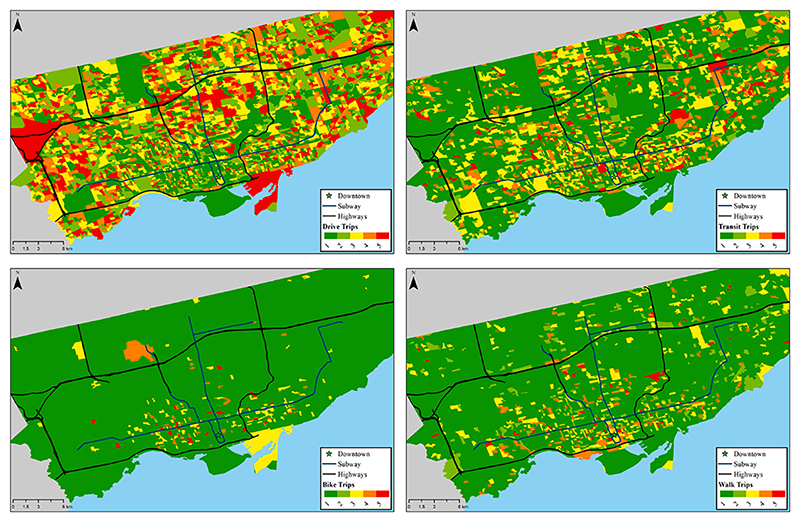
Map of Greater Toronto region with modal trip frequencies, clockwise (from top right) the levels of transit, walk, bicycle and auto trips (green for lowest category 1, up to red for highest category 5). (For interpretation of the references to colour in this figure legend, the reader is referred to the web version of this article.)

For auto trip frequency, zero trips per household is considered to be in the very low category, between one and two trips is in the low category, between three and four trips are in the medium category, between five and seven trips are in the high category, and eight or more trips is in the very high category. For transit, bicycle and walk trip frequencies, zero trips per household is in the very low category, one trip is in the low category, two trips are in the medium category, between three and four trips are in the high category, and five or more trips are in the very high category. For auto, transit, bicycle and walk access, the continuous value distribution obtained from the accessibility calculation is divided into very low, low, medium, high, and very high categories by using the 20%, 40%, 60%, and 80% quantiles. To facilitate a broader understanding of the study area, and to provide empirical understanding of the Toronto region, the modal trip categories and accessibility categories are overlayed on Toronto city map in Figs. 1 and 2. Fig. 1 shows a map of Greater Toronto region with modal accessibilities, clockwise (from top right in the figure) the levels of transit, walk, bicycle and auto accessibility (green for lowest category 1, up to red for highest category 5). Similarly, Fig. 2 shows a map of Greater Toronto region with modal trip frequencies, clockwise (from top right) the levels of transit, walk, bicycle and auto trips (green for lowest category 1, up to red for highest category 5). For transit mode, we can observe that while the south-central region provides highest levels of accessibility, the trip distribution is spread throughout the region, with no clear central transit trip demand. The east and the west sides of the region represent lowest levels of transit accessibility, with non-zero levels of transit trips observed. For walk and bicycle modes, the accessibility representation shows multiple high-level zones, however, with very low levels of trips made by these modes. Lastly, for the auto mode, the zones with high levels of accessibility are evenly spread throughout the region, illustrating how infrastructure is tilted towards auto mode choice. However, only south-central region in the map can be shown to have minimal number of auto-trips, as represented by a higher proportion of the green. To summarise, auto-supportive infrastructure is available throughout the Toronto metropolitan region, providing higher levels of auto accessibility. On the other hand, the transit and bicycle networks primarily serve the city-centre regions, with lower level of accessibility by transit and bicycle in the outer urban and suburban neighbourhoods.

## 3 Study methodology

For the household trip frequencies by each mode, the multivariate model system used in this paper assumes an underlying set of multivariate continuous latent variables whose partitioning maps into the observed set of count outcomes. Based on the observed data, these count outcomes are translated into ordered categories of low, medium, and high trip counts. Examples of cases where the ordered-response system is used to model count outcomes include household car ownership levels (Kitamura, 1988; Bhat and Guo, 2007) and trip generation/stop-making (Meurs, 1989; Agyemang-Duah et al., 1995; Agyemang-Duah and Hall, 1997; Carrasco and Miller, 2009). For the accessibility for each mode outcome, the observed continuous outcomes were also mapped into ordered categories.^1^

Thus, this joint model system can be estimated using a multivariate ordered-response framework. The ordered-response system allows the use of a general correlation matrix for the underlying latent variables, which translates to a flexible correlation pattern among the observed outcomes. Even though the ordered-response models are used for ordinal responses, the distinction between ordinal and cardinal responses is irrelevant for modelling purposes.^2^ This is valid in the cases where the count responses are finite and have few discrete values as in the current empirical application. To avoid the convergence problems associated with estimating a joint multivariate ordered-response system with a large number of categories, we use the composite marginal likelihood technique (Varin and Vidoni, 2005; Varin, 2008).

This paper considers the eight-variate choice system as a bundled choice and explores the inter-relationships among them, without imposing a structure a priori wherein the accessibility is determined first and then exogenously affects the modal trip frequencies. This effort is motivated by the desire to seek answers to the questions described in section 1 where we aim to understand the directionality, type, and extent of effect between the variables of interest. An exploration of the inter-relationships between the accessibility by mode and trip frequencies by mode would help advance an understanding of how the availability (or the lack thereof) of certain modes influences trip making decisions and whether higher accessibility by certain modes is a precise criterion for travellers’ mode choice. This section presents the methodological approach used in this study. The model formulation (Multivariate Ordered Probit model) is presented first; this is followed by a description of the conceptual framework used to explore inter-outcome dependencies.

### 3.1 Model formulation

Let *q* be an index for households, *i* be the index for ordered response category (where *I* denote the total number of dependent categories for each individual) and number of ordered response values for category *i* be *Ki* + 1, such that:

(1) q=1,2,…,Q;hereQ=42,718

(2) i=1,2,…,I;inthecurrentstudy,I=8

(3) k=0,1,2,…,Ki;

where, the discrete levels k, belong in *{0, 1, 2, …, Ki}* for category *i*.

This model found its origin in biostatistics and social sciences (Aitchison and Silvey, 1957; McKelvey and Zavoina, 1975) long before it was used for applied econometric studies (Lesaffre and Molenberghs, 1991; Kim, 1995). We can write the latent propensity $\left(y_{qi}^{*}\right)$ for each ordered response as a function of its covariates and this latent propensity can be translated into the observed outcome (*y*_*qi*_) through threshold bounds:

(4) $$y_{qi}^{*}=\beta_{i}^{\prime }x_{qi}+\varepsilon_{qi},y_{qi}=kif\theta_{i}^{k}<y_{qi}^{*}<\theta_{i}^{k+1}$$

where, *x*_*qi*_ is a (*L* × 1) vector of exogenous variables (*L* does not include a constant), *β*_*i*_ is a corresponding (*L* × 1) vector of coefficients to be estimated, *ε*_*qi*_ is a standard normal error term, and $\theta_{i}^{k}$ is the lower bound threshold for count level *k* of ordered response category *i* $(\theta_{i}^{0}<\theta_{i}^{1}<\theta_{i}^{2}\ldots <\theta_{i}^{K_{i}+1};\theta_{i}^{0}=-\infty ,\theta_{i}^{K_{i}+1}=+\infty$ for each category *i*). The *ε*_*qi*_ terms are assumed independent and identical across individuals (for each and all *i*). For identification reasons, the variance of each *ε*_*qi*_ term is normalised to 1, to correctly identify and estimate the threshold values. However, we allow correlation in the *ε*_*qi*_ terms across ordered response categories *i* for each individual *q*.

Specifically, define *ε*_*q*_ = (*ε*_*q*1_, *ε*_*q*2_, *ε*_*q*3_, ⋯, *ε*_*qI*_)′. Then, *ε*_*q*_ is multivariate normal distributed with a mean vector of zeros and a correlation matrix as follows:

(5) $$\varepsilon_{q}\sim N\left[\left(\begin{array}{c} 0\\ 0\\ \vdots \\ 0 \end{array}\right),\left(\begin{array}{ccccc} 1 & \rho_{12} & \rho_{13} & \cdots & \rho_{1I}\\ \rho_{21} & 1 & \rho_{23} & \cdots & \rho_{2I}\\ \vdots & \vdots & \vdots & \ddots & \vdots \\ \rho_{I1} & \rho_{I2} & \rho_{I3} & \cdots & 1 \end{array}\right)\right],\;\text{or}\;$$

*ε*_*q*_~*N*[0, **Σ**]

The off-diagonal terms of Σ capture the error correlations across the underlying latent continuous variables of the different ordered response categories; that is, they capture the effect of common unobserved factors influencing the propensity of choice of count level for each ordered response. Thus, if *ρ*_12_ is positive, it implies that households with a higher-than-average propensity in their data sample towards the first ordered response variable are also likely to have a higher-than-average propensity towards the second ordered response variable. Of course, if all the correlation parameters (*i.e*., off-diagonal elements of Σ), are identically zero, the model system in Equation (4) collapses to independent ordered response probit models for each ordered response.

Let the actual observed count level for household *q* and ordered response category *i* be *m*_*qi*_. Then, the likelihood function for household *q* may be written as follows:

(6) $$L_{q}(\delta )=Pr\left(y_{q1}=m_{q1},y_{q2}=m_{q2},\ldots ,y_{qI}=m_{qI}\right)$$

(7) $$L_{q}(\delta )={\int \;}_{v_{1}=\theta_{1}^{m_{q1}}-\beta_{1}^{\prime }x_{q1}}^{\theta_{1}^{m_{q1}+1}-\beta_{1}^{\prime }x_{q1}}{\int \;}_{v_{2}=\theta_{2}^{m_{q2}}-\beta_{2}^{\prime }x_{q2}}^{\theta_{2}^{m_{q2}+1}-\beta_{2}^{\prime }x_{q2}}{\int \;}_{v_{I}=\theta_{I}^{m_{qI}}-\beta_{I}^{\prime }x_{qI}}^{\theta_{I}^{m_{q}I+1}-\beta_{I}^{\prime }x_{qI}}\varphi_{I}\left(v_{1},v_{2},\ldots ,v_{I}|\Sigma \right)dv_{1}dv_{2}\ldots dv_{I}$$

where *φ*_*I*_(*v*_1_, *v*_2_, …, *v*_*I*_|**Σ**) represents the multivariate normal density of dimension *I* with correlation matrix **Σ**, evaluated at the abscissae (*v*_1_, *v*_2_, …, *v*_*I*_). In order to overcome the computational complexity involved in calculating high-order *I*-dimensional rectangular integral, this study employs a Composite Marginal Likelihood (CML) computation approach, which involves approximating the higher-order integral through the computation of a series of bivariate marginal distributions; the CML estimator (theoretically speaking) loses some accuracy relative to traditional maximum likelihood estimation, though this efficiency loss has been showed to be negligible in practice (Varin and Vidoni, 2005). The model is estimated using R ‘mvord’ package (Hirk et al., 2017), MASS package (Venables and Ripley, 2013)and Python software written by the authors.

### 3.2 Conceptual framework

The multivariate ordered probit modelling methodology is used to analyse the influence of exogenous variables on the eight outcome variables of accessibility levels and household trip frequencies and to explore the nature of the relationships among them. As noted earlier, the eight endogenous variables represent the four accessibility levels (one for each mode) and four trip-counts:

- Accessibility by auto, labelled as “Auto Acc”
- Accessibility by transit, labelled as “Tr Acc”
- Accessibility by bicycle, labelled as “Bicycle Acc”
- Accessibility by walk, labelled as “Walk Acc”
- Trip frequency by auto, labelled as “Auto Trips”
- Trip frequency by transit, labelled as “Tr Trips”
- Trip frequency by bicycle, labelled as “Bicycle Trips”
- Trip frequency by walk, labelled as “Walk Trips”

**Fig. 3:**
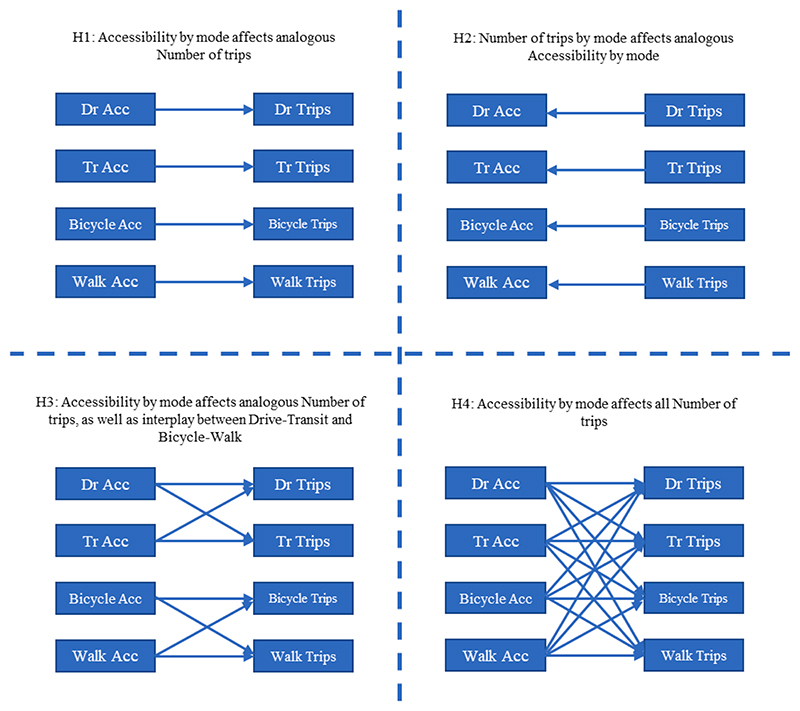
Hypotheses Regarding Relationships Among Endogenous Variables.

The presence of the eight endogenous variables gives rise to many possible specifications of relationships among them. With respect to the myriad of relationships among the endogenous variables, four model structures were considered. These are depicted in Fig. 3 (as H1 through H4). They include:

- H1: Accessibility by mode affects corresponding trip frequency
- H2: Trip frequency by mode affects corresponding accessibility
- H3: Accessibility by mode affects corresponding trip frequency with added interplay between Auto-Transit accessibility affecting each-other’s trip frequency and bicycle-walk accessibility affecting each-other’s trip frequency
- H4: Accessibility by each mode affects all modal trip frequencies

In all specifications, an error correlation structure is incorporated to accommodate the bundled nature of the many choices. Thus, the directionality of the relationship of an observed endogenous variable on the propensity underlying another endogenous variable can only be in one direction. The reader will note that a recursive endogenous effect of one observed ordinal variable on the propensity of the other observed ordinal variable is possible in the equation system, although including both directions of observed effects lead to a logically inconsistent model structure because the probabilities of all the possible combinations of discrete observations will not sum to one (see Maddala, 1983 and (Maddala, 1986)). Intuitively, the propensities are the precursors to the actual observed variables, and, when both the decisions are co-determined, it is impossible to have both observed variables structurally affect one another. In the current estimation effort, all models with every possible impact direction were estimated and the best fit version was chosen. However, regardless of the directionality between endogenous outcomes and its significance, the entire model system is a joint bundle of choices because of the correlation in the unobserved factors affecting underlying propensities. After estimating model specifications corresponding to H1 through H4, we used goodness-of-fit measures, behaviour intuitiveness, and statistical significance of coefficients to arrive at a final model structure that was both intuitive and supported by the data. The final model structure is based on the hypothesis H4, which provides the highest goodness-of-fit and incorporates all cross-modal endogenous effects between accessibility and trip frequency. Models H1 and H3 were building blocks to the model H4, while H2 had no statistically significant endogenous effects. Thus, in addition to being contextually dependent, the results presented in this paper are fundamentally empirical.

## 4 Model estimation results

The final model structure is based on the hypothesis H4. The results shown here are based on statistical significance, providing inferential information about the given empirical context. Additional evaluations of probability of correct predictions offered by the model are suppressed in the interest of brevity. Essentially, both jointness (due to error correlations) as well as directional effects need to be accommodated. Finally, in the remaining sections we discuss the important results for each of the household structure groups and their interpretations. As mentioned before, the household structure models are estimated independently for single adult with no children, adult couple with no children, single or couple with one or more children, multi-adult with no children, and multi-adult with one or more children.

For brevity, this section focusses entirely on the results for “couple adult with no children” household type for the hypothesis H4, which represents household with two adults over the age of 16 years living together and no children. We have chosen this household structure as it constitutes a greater proportion of total households in the region. While the results focus on this singular category, the findings of other household structure estimations are similar and are available in ‘Appendix A’.

### 4.1 Exogenous Variable Effects

Tables 3 and 4 present estimation results depicting the influence of the exogenous variables on the various modal trips and accessibilities, respectively, for the multivariate models for each of the structures with best goodness-of-fit. While the modal trip frequencies are directly observed in the data set (in the form of household trips and primary mode for each trip), the accessibility values are calculated as detailed in the earlier sections. Dummy variables for presence of people less than 55 years of age are found to increase transit trip frequencies significantly, as compared to those above 55 years of age. Transit trip frequencies are almost equally influenced by people in all age-groups, with a slightly higher coefficient observed for households with young adults. This may be due to the tendency of young couples to prefer train and bus services, coupled with lower affordability of cars and self-selection into neighbourhoods closer to city centre. The propensity to engage in driving trips is lower with presence of younger individuals in the household, while higher driving was observed with presence of 56 years of age or older. Lower bicycle trip frequencies are associated with households with people in the age-group 66 and above, which can be attributed to the fact that riding a bicycle can be risky and requires higher agility and fitness levels. Similarly, lower bicycle trip frequency is observed for individuals in the age group 16–25 which includes high-school and university students. This could be attributed to either preference for driving to school or for university students the impact of living close to campus. This is further attributed by a higher walk trip coefficient for this age group suggesting more walk trips, probably due to living on-campus or closer to central zones. We observe a negative association of walk trips with age group 66 years and above. Our results are similar to those observed by earlier research focusing on Canadian individuals showing that younger people are more likely to walk, bike, or use transit (Newbold and Scott, 2017; Newbold and Scott, 2018); and that younger people have a higher level of multimodality compared to older adults (Sakaria and Stehfest, 2013). In contrast, older adults exhibit a decrease in the probability of using transit as they age (Moniruzzaman et al., 2013). It has been suggested that within older adults, older seniors are resistant to adopting alternative transportation modes, whereas younger seniors show an increasing trend in the use of alternative forms of transport (Fordham et al., 2017). Reasons for this resistance may include unfamiliarity with the service, lack of habituation, and limited knowledge of the required technology, such as smartphone applications (Fordham et al., 2017).

Presence of students in the household has a positive association with auto, transit and walk trip frequencies, with highest coefficient observed for transit trips which can be attributed to the fact that students often live on a budget and benefit from incentives to use public transport which induces a preference towards the mode. Our results are in line with Brown et al. (2016), who conclude that younger adults were more likely to ride transit than older adults due to life cycle factors such as being a student. Distance to CBD (measured in kms) is increased auto and transit trip frequencies and decreased bicycle and walk trip frequencies. The results presumably indicate the presence of more bicycle and walk friendly neighbourhoods close to downtown areas, while as we move away from the CBD, driving is preferred to bicycle and walk modes due to the increasing distance between destinations and lack of cycling and walking infrastructure. The positive parameter for transit trips for distance to the CBD is also consistent with the observed levels of transit trips as shown in Fig. 2. Contrary to expectations, more transit trips can be observed in regions surrounding the city-centre, with few much farther from the CBD. The accessibility by each mode is negatively associated with the distance to CBD, denoting that the extent of employment opportunities reachable reduces as we move further away from the CBD. This trend can be seen in cities other than Toronto, as evident in results by Moniruzzaman et al., (2015) who found that distance to CBD reduces walking trips but increases vehicle trips in Montreal.

Higher population density is associated with lower auto and bicycle trips but higher walk trips which may be attributed to the fact that higher density neighbourhoods are often more congested and may have less driver-friendly infrastructure. On the other hand, higher population density is associated with higher auto and transit accessibilities which suggests that the amount of employment opportunities by each of these modes is higher in regions where there is a greater density of settlement. Higher bicycle accessibility was found in areas of greater employment density indicating that the cycling infrastructures are better in areas with higher employment density. This is consistent with Faghih Imani et al. (2019) who found that there are often not enough available low-stress links for a 30-minute isochrone, with high stress roads and lack of cycling infrastructure decreasing low-stress accessibility by space limitations rather than the cut off time. Further, as expected, higher employment density is associated with higher transit accessibility and lower auto access. However, we observe a counterintuitive negative association with walk accessibility.

High-income households are more likely to engage in driving trips, consistent with their greater vehicle ownership and ability to afford costs associated with driving. The coefficients increase each increase in income. Lower-income households are also observed to make fewer transit trips than their higher-income counterparts. Lower trip frequencies observed in lower income groups have been attributed to time-poverty which restricts the extent of non-mandatory activities that lower income group households can participate in, and equity issues regarding the distribution of transport infrastructure in Toronto (Allen and Farber, 2020). Results from California (Boarnet et al., 2020) indicate that while high-income households do make a larger number of trips, there may be a possibility of reduction in vehicular travel for residences very close to transit stations. However, they find no evidence of any income differences in changes in the propensity to use transit when living near versus far from a transit station. This conclusion is particularly fascinating as it bolsters our conclusion regarding neighbourhood dissonance of transit riders. Accessibility to opportunities by transit were negative for all income groups, signalling a need for increasing overall transit accessibility. However, higher income households have a slight advantage as compared to lower-income counterparts as they may reside in residential locations which have better public transport infrastructure and lower level of traffic stress for reaching a higher number of employment opportunities. Accessibility by bicycle increases with income level as it can be estimated that higher income neighbourhoods provide better bicycling facilities and an overall better built-environment infrastructure. We observe a higher propensity to transit trips and a lower propensity to bicycle trips for households which have a higher fraction of female adults, consistent with previous findings (Mitra et al., 2016; Imani et al., 2019). Vehicle availability per licensed adult is found to significantly impact all modal trip frequencies. Greater than zero vehicle availability is associated with a higher number of auto trips, and a lower number of transit, bicycle and walk trips, as compared to household with zero vehicle availability. This is consistent with the expectation that higher auto availability facilitates driving trips and inhibits trips by other modes of transport.

However, we observe a lower accessibility to opportunities by driving and transit for households with vehicles. Vehicle owners may reside in regions which are further away from locations of employment opportunities and may be willing to drive longer distances; or some low-income people without vehicles may reside in suburban regions in Toronto with very high auto access. We also observe lower transit accessibility for households with more than one vehicle available per licensed adult. Transit pass ownership significantly impacts driving negatively and transit trip frequencies positively, intuitively suggesting that a higher transit pass ownership suggests that a household makes higher transit trips and lower trips by other modes of transport. Households with workers show higher trip frequencies as compared to household with no workers. Dual-worker households are more likely to participate in fewer activities than single-worker households, which could be due to both members participating in multiple mandatory and non-mandatory trips. Finally, housing type significantly impacts auto, transit and bicycle trips. Apartment dwellers drive and cycle less but make more transit trips. This can be attributed to the fact that apartment dwellers usually have a lower vehicle ownership as compared to house or townhouse owners and usually reside in transit-rich neighbourhoods. Apartment dwellers also have a lower auto and walk accessibility which may refer to a distribution of non-egalitarian walk-friendly facilities. Empirically, the City of Toronto does have a huge number of high-rise apartment buildings in the suburban neighbourhoods that generally have relatively lower non-motorised accessibility, as compared to urban neighbourhoods closer to city centre.

### 4.2 Endogenous Variable Effects and Correlation Effects

**Fig. 4:**
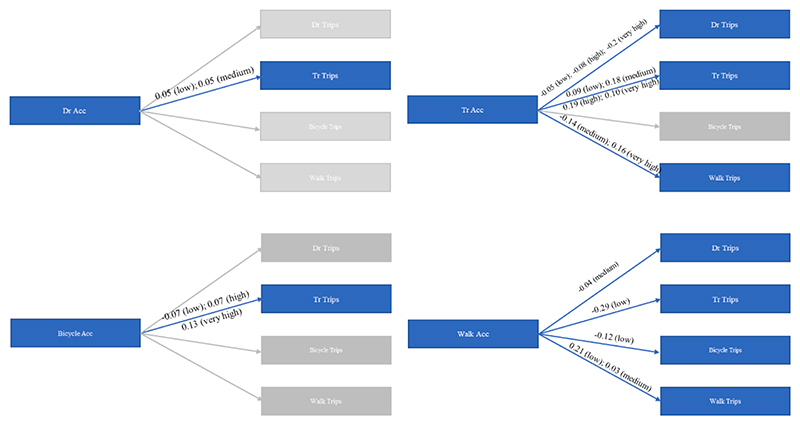
Endogenous Variables Effects (the highlighted blue are the significant effects, with values signifying the coefficients, and ‘low/medium/high/very high’ in parenthesis signify the level of accessibility for which the effect is quantified. (For interpretation of the references to colour in this figure legend, the reader is referred to the web version of this article.)

A key objective of this study is to unravel the complex interplay of relationships among the eight endogenous variables with a view to better understand the complementarity and substitution effects that may be present. Table 5 offers estimates of the endogenous variable effects, while Table 6 offers goodness of fit together with error correlations. A graphical depiction of the significant endogenous variable effects retained in the final model specification is shown in Fig. 4 (specification H4 with only significant relationships). Note that these effects are “true” direct effects because the jointness among the eight endogenous variables has been captured through the error correlations.

There is a positive effect of higher auto accessibility (significant only at 90 % confidence interval, as compared to very low auto access) on transit trips. Indeed, a higher accessibility to opportunities by driving may be indicative of an outcome of the neighbourhood’ s central location within the metropolitan area. This is an interesting finding which hasn’t been reported earlier for lack of multi-modal multivariable model estimations. However, higher transit accessibility reduced auto trips and increased transit trips. The complementarity between auto accessibility and transit trips and the substitution effect observed between transit accessibility and auto trips may hint towards the fact that providing greater transit facilities with better connectivity and employment reach can positively impact the desired reduction in driving trips. Further, it also highlights the residential neighbourhood dissonance of transit riders and shows that location with higher transit accessibility (that is better transit service) are not necessarily locations where people who make more transit trips reside. The lack of good transit service may be the reason that some people have to make more transit trips to reach their destinations. Very high transit accessibility also positively impacts walk trips. This may be because residences in transit rich neighbourhoods may also have accessibility to other commercial developments nearby, thereby causing a higher number of walk trips.

We observe a complementary relationship for bicycle accessibility on transit trips. Higher bicycle accessibility has a positive impact on transit trips. This could indicate towards the fact that a bicycle friendly neighbourhood may also be transit rich, thereby leading to more transit trips. This, however, this depends on the level of stress associated with biking, which may vary from location to location, and certain neighbourhoods with much lower levels of stress from traffic are predominantly transit rich neighbourhoods. Similarly, we observe walk accessibility to reduce auto, transit, and bicycle trips. If the neighbourhood is less walk accessible (and by extension, often also less transit- and bike-accessible) then it will lead to more car trips.

The error correlations in the bottom of Table 6 show that several error covariances are statistically significant in this dataset. This is to be expected, as unobserved household socio-economic and demographic attributes are likely to simultaneously affect trip frequencies and modal accessibilities. Mostly negative correlations between modal trip frequencies suggest that the unobserved attributes that motivate the use of any one mode choice, inhibit the use of all other mode types. The exception being the positive correlation between bicycle and walk trips which shows that unobserved factors that support either type of active transport trips leads to similar preference for the other active mode. We observe a highly significant positive correlation between auto accessibility and walk access.

However, a negative correlation between transit-accessibility and walk-accessibility is observed which could be the result of unobserved characteristics favouring one or the other. There are also a few significant correlations between accessibility-trip combinations of endogenous variables. While the observed attributes resulted in a direct negative effect of transit accessibility on auto trips, the unobserved attributes lead to a positive correlation between the two. Also, the observed attributes resulted in a positive direct impact of auto accessibility on transit trips, the unobserved attributes lead to a negative correlation between the two. And the observed attributes resulted in a positive direct impact of transit accessibility on transit trips, the unobserved attributes lead to a negative correlation between the two. We observe a highly significantly negative correlation between auto and transit accessibilities, and a significantly positive correlation between auto and walk accessibilities. A positive correlation between transit and bicycle accessibilities could also be attributed to the fact that unobserved factors leading to better cycling facilities are predominantly transit rich neighbourhoods. However, a negative correlation between transit and walk accessibilities is observed which could be the result of unobserved characteristics favouring one or the other. There are also a few significant correlations between accessibility-trip combinations of endogenous variables. The observed attributes resulted in a positive direct impact of transit accessibility on transit trips, the unobserved attributes lead to a negative correlation between the two.

These results bolster the importance of a multivariate ordered probit modelling methodology as there are significant error correlations that capture the effects of correlated unobserved attributes simultaneously affecting multiple trip frequency and accessibility outcomes. It is imperative that these error correlations be estimated so that consistent estimates of exogenous and endogenous variable effects can be obtained, and accurate forecasts of combinations of the eight endogenous variables may be made.

## 5 Conclusions

This paper presents a joint modelling framework that simultaneously models the employment accessibility and household trip frequency by different transport modes using data for the City of Toronto. Accessibility measures were generated in the form of cumulative opportunities to employments within an isochrone for the four travel modes (auto, transit, bicycle, and walk). Household trip frequencies were calculated for each of the four travel modes by aggregating the total number of trips made by the household members by that mode. Both trip frequencies and accessibility measures were then categorised in ordered levels of low, medium, and high. The paper employs a multivariate ordered probit model system allowing dependence between the trip frequencies by different modes and accessibility by the modes due to both observed and unobserved factors. The modelling framework is used to analyse the influence of exogenous variables on the eight outcome variables of accessibility levels and trip frequencies and to explore the nature of the relationships among them. The results confirm our hypothesis that not only does a strong association exist between employment accessibility and household trip frequency, but there are direct effects to be observed. Separate models were estimated for different household structures such as single adult with no children, adult couple with no children, single or couple with one or more children, multi-adult with no children, and multi-adult with one or more children. The results section provided inferences about “adult couple with no children” household type, which represents household with two adults over the age of 16 years living together and no children.

In addition to observed direct effects, there are significant unobserved effects (error correlations) that simultaneously affect multiple trip frequency and accessibility outcomes. Ignoring such effects would result in inconsistent estimates of exogenous and endogenous variable effects. The endogenous variable effects are of key interest in this study. The final model structure that provides the best and most intuitive results shows that there are both complementary and substitution effects at play. We observe a complementarity effect between auto accessibility and transit trips, and a substitution effect observed between transit accessibility and auto trips. Based on these results, policies oriented towards providing greater transit facilities with better connectivity and employment reach can positively impact the desired reduction in driving trips. Further, it also highlights the residential neighbourhood dissonance of transit riders and shows that locations with higher transit accessibility (that is, better transit service) are not necessarily locations where people who make more transit trips reside. People who are fairly transit dependent (e.g., low-income people and/or people without cars) need to take transit a lot, despite a lack of good transit access. For future transit-development policymakers can utilize our results to identify the transit-poor neighbourhoods within Toronto and improve transit facilities. Such policy measures can have a multi-fold advantage. A targeted transit development can provide a higher accessibility to low-income neighbourhoods, but also might impact vehicular congestion on the streets of Toronto.

Further, the results demonstrate that on average, older households in the sample tend to live in suburban neighbourhoods that are auto-oriented, while younger households tend to live in more “urban” (aka downtown) walk/transit-oriented neighbourhoods. We also observe a preference for auto as one moves further away from the downtown. Suburbanization and its effects on variability of modal preferences is very well reflected in the results such that driving is preferred to bicycle and walk modes due to the increasing distance between destinations and lack of cycling and walking infrastructure. The limiting bicycle infrastructure in Toronto are better placed for the locations with higher employment density which does not serve the population at large and disincentivises trip utility for higher population density neighbourhoods. We are also able to identify what are potentially time impoverished population groups, restricting the extent of activities that they can participate in and raising an equity issue regarding the distribution of transport infrastructure in Toronto.

In summary, the study’s findings both affirm existing research and challenge conventional notions, while also introducing entirely new insights into travel behaviour and accessibility. In agreement with previous findings, younger adults exhibit a preference for transit, walking, and biking, while students tend to rely more on transit for their trips. Income levels significantly influence driving trips, with higher-income households demonstrating a greater propensity for driving. However, challenging previous findings, our results reveal that transit trips do not consistently decrease as one moves farther from the Central Business District (CBD). High bicycle accessibility can encourage transit trips, defying traditional assumptions. The novel outcomes of this analysis include a previously unexplored complementarity between auto accessibility and transit trips, suggesting that households with better access to private vehicles may reside in areas facilitating a higher number of public transit trips. Conversely, a substitution effect is observed between transit accessibility and auto trips, indicating that improving transit options could decrease the need for private vehicle use. The results highlight the residential dissonance of transit riders, as locations with high transit accessibility may not necessarily align with where frequent transit users reside. Furthermore, it underscores that improved walk accessibility primarily promotes active trips while reducing the use of other modes.

The analysis is not without limitations. While we account for observed attributes obtained from this empirical data, future development of similar analysis can benefit from attitudinal information, which could also be supported by an integrated choice and latent variable (ICLV) model framework. More comprehensive and multi-dimensional accessibility indicators may provide a more realistic substitute for the plethora of built environment (or even residential location choice), since residential location or relocation is often caused by attractive employments, education and healthcare opportunities and better lifestyle. Similarly, accessibility measures for walk accessibility may consider the “quality” of the walking experience. Also, trip frequencies might be usefully categorised by purpose. The TTS data has also been observed to under-report walk and bicycle trips and short distance trips (Harding et al., 2018).

## Supplementary Material

Appendix

## Figures and Tables

**Table 1:** Description of Sample (n = 42,718).

Independent (continuous) Variables Distribution Variable	Mean	Sth. Dev.	Min	Max
Fraction of female adults in the households	0.54	0.32	0.00	1.00
Fraction of male adults in the households	0.46	0.32	0.00	1.00
Distance to CBD (km)	10.18	6.05	0.00	26.77
Population Density (persons per sq. km)	14,656.68	19,416.81	0.00	240,911.54
Employment Density (persons per sq. km)	6,412.27	21,079.68	0.00	263,070.47
Population Density (Standardised value*)	0.00	1.00	−0.75	11.65
Employment Density (Standardised value*)	0.00	1.00	−0.30	12.18

* Created by subtracting the mean from each value and dividing by the standard deviation (same as *z scores*).

**Table 2:** Number of observations (%) of trips by mode for each category of trip frequency (n = 42,718).

	Very Low	Low	Medium	High	Very High
Auto Trips	15,588 (36.5)	9,945 (23.3)	7,771 (18.2)	5,404 (12.7)	4,010 (9.4)
Transit Trips	24,588 (57.6)	1,965 (4.6)	10,139 (23.7)	4,425 (10.4)	1,601 (3.7)
Bicycle Trips	40,568 (95.0)	110 (0.3)	1,276 (3.0)	534 (1.3)	230 (0.5)
Walk Trips	34,327 (80.4)	2,097 (4.9)	4,026 (9.4)	1,722 (4.0)	546 (1.3)

**Table 3:** Exogenous Variable Effects on Modal trip frequencies (“-” is used for insignificant parameter estimates).

Variable	Auto Trips			Transit Trips			Bicycle Trips			Walk Trips	
	Estimate	z value		Estimate	z value		Estimate	z value		Estimate	z value
*(Base: Age category 16–25 years)*											
Presence in age category 26–35 years	–0.1034	–3.86		0.2355	7.80		–	–		–	–
Presence in age category 36–45 years	–0.0531	–1.93		0.1625	5.43		–	–		–	–
Presence in age category 46–55 years	–0.0788	–3.14		0.1501	5.16		–	–		–	–
Presence in age category 56–65 years	0.1172	4.34		–	–		–	–		–	–
Presence in age category 66 and above years	–	–		–	–		−0.4601	−7.62		−0.1022	−2.62
Presence of students in the household	–	–		0.2819	8.37		0.3018	5.58		0.1721	4.04
Distance to CBD (km)	0.0113	4.88		0.0118	4.07		−0.0931	−21.47		−0.0705	−22.97
Population Density (Standardised value)	−0.0291	−2.77		–	–		−0.0973	−4.49		0.0450	3.42
Employment Density (Standardised value)	−0.0208	−2.18		−0.0526	−5.06		−0.1252	−6.13		0.0683	6.23
*(Base: Income less than 15,000$)*											
Income 15,000$-40,000$	–	–		0.0933	1.56		–	–		–	–
Income 40,000$ to 60,000$	0.1997	6.38		0.1084	1.76		–	–		–	–
Income 60,000$ to 100,000$	0.3342	11.29		0.2287	3.85		–	–		–	–
Income 100,000$ to 125,000$	0.4032	11.51		0.3286	5.18		–	–		–	–
Income greater than 125,000$	0.4907	15.35		0.3162	5.08		–	–		–	–
*(Base: fraction of male adults in the household)*											
fraction of female adults	–	–		0.1909	3.94		−0.2627	−2.94		–	–
*(Base case: Vehicle availability per licensed adult (VAPLA) is zero)*											
VAPLA is between zero and one	1.3931	42.01		−0.5656	−19.43		−0.4609	−10.46		−0.2780	−8.47
VAPLA as one or more	1.6123	47.18		−1.0136	−33.66		−0.9081	−17.11		−0.5488	−15.76
*(Base case: Transit pass ownership per adult (TPOPA) is less than one)*											
TPOPA one or more	−0.3582	−12.79		0.7115	26.88		−0.2557	−5.86		–	–
*(Base case: No worker household)*											
Single worker household	–	–		0.4793	14.27		–	–		0.2339	5.44
Multi-worker household	0.1170	5.05		0.5985	18.12		0.2250	4.60		0.3034	6.90
*(Base case: dwelling type house or townhouse)*											
Apartment type dwelling	−0.1020	−4.72		0.1426	5.83		−0.3216	−7.21		–	–

**Table 4:** Exogenous Variable Effects on Modal Accessibilities (“–” is used for insignificant parameter estimates).

Variable	Auto Accessibility			Transit Accessibility			Bicycle Accessibility			Walk Accessibility	
	Estimate	z value		Estimate	z value		Estimate	z-value		Estimate	z value
*(Base: Age category 16–25 years)*											
Presence in age category 26–35 years	–	–		–	–		−0.1074	−5.25		–	–
Presence in age category 36–45 years	–	–		–	–		−0.0886	−3.71		–	–
Presence in age category 46–55 years	–	–		–	–		–	–		–	–
Presence in age category 56–65 years	–	–		–	–		–	–		–	–
Presence in age category 66 and above	–	–		–	–		–	–		–	–
Presence of students in the household	–	–		–	–		–	–		–	–
Distance to CBD (km)	−0.0133	−7.91		−0.1852	−80.81		−0.0838	−47.01		0.0207	13.34
Population Density (Standardised value)	0.2365	25.66		0.2489	24.69		–	–		–	–
Employment Density (Standardised value)	−2.1572	−112.71		0.4428	44.02		0.1059	13.24		− 0.1416	−15.58
*(Base: Income less than 15,000$)*											
Income 15,000-40,000$	0.1358	5.51		−0.1255	−2.31		–	–		0.0970	4.08
Income 40,000$ to 60,000$	–	–		−0.1140	−2.07		0.1042	3.39		–	–
Income 60,000$ to 100,000$	–	–		−0.1124	−2.11		0.1244	4.49		–	–
Income 100,000$ to 125,000$	–	–		−0.1576	−2.81		0.1408	4.36		–	–
Income greater than 125,000$	–	–		−0.0951	−1.76		0.2282	8.09		–	–
*(Base: fraction of male adults in the household)*											
fraction of female adults	–	–		–	–		0.1029	2.33		–	–
*(Base case: Vehicle availability per licensed adult (VAPLA) is zero)*
VAPLA is between zero and one	−0.0866	−3.57		−0.0716	−2.45		–	–		–	–
VAPLA as one or more	−0.0794	−3.35		−0.1735	−5.95		–	–		–	–
*(Base case: No worker household)*											
Single worker household	–	–		0.0531	2.39		–	–		–	–
Multi-worker household	–	–		–	–		–	–		–	–
*(Base case: dwelling type house or townhouse)*											
Apartment type dwelling	−0.6051	−28.92		–	–		−0.1321	−6.84		−0.4568	−24.07

**Table 5:** Endogenous Variable Effects (“-” is used for insignificant parameter estimates).

Variable	Auto Trips			Transit Trips			Bicycle Trips			Walk Trips	
	Estimate	z value		Estimate	z value		Estimate	z value		Estimate	z value
*(Base case: Auto Access Very Low)*											
Auto Access Low	–	–		0.0539	1.95		–	–		–	–
Auto Access Medium	–	–		0.0521	1.87		–	–		–	–
Auto Access High	–	–		–	–		–	–		–	–
Auto Access Very High	–	–		–	–		–	–		–	–
*(Base case: Transit Access Very Low)*											
Transit Access Low	−0.0510	−1.99		0.0967	2.60		–	–		–	–
Transit Access Medium	–	–		0.1826	4.91		–	–		−0.1469	−3.71
Transit Access High	−0.0832	−2.80		0.1913	4.63		–	–		–	–
Transit Access Very High	−0.2576	−7.14		0.1081	2.32		–	–		0.1610	4.35
*(Base case: Bicycle Access Very Low)*											
Bicycle Access Low	–	–		−0.0682	−2.23		–	–		–	–
Bicycle Access Medium	–	–		–	–		–	–		–	–
Bicycle Access High	–	–		0.0742	2.56		–	–		–	–
Bicycle Access Very High	–	–		0.1351	4.56		–	–		–	–
*(Base case: Walk Access Very Low)*											
Walk Access Low	–	–		−0.1940	−6.79		−0.1271	−2.54		0.2120	6.24
Walk Access Medium	−0.0466	−1.99		–	–		–	–		0.0368	1.06
Walk Access High	–	–		–	–		–	–		–	–
Walk Access Very High	–	–		–	–		–	–		–	–

**Table 6:** Correlation Effects and Goodness-of-fit.

	Auto Trips			Transit Trips			Bicycle Trips			Walk Trips	
	Estimate	z value		Estimate	z value		Estimate	z value		Estimate	z value
*Thresholds*											
Intercept 1|2	0.9848	19.53		1.0427	12.29		0.0686	0.85		0.4225	7.17
											
Intercept 2|3	1.7602	34.12		1.1854	13.97		0.1098	1.35		0.6582	11.10
Intercept 3|4	2.4563	46.93		2.1584	25.33		0.6315	7.59		1.3287	22.46
Intercept 4|5	3.1817	59.84		3.1570	35.97		1.1619	13.20		2.1287	33.62
*Correlation Terms*											
Auto Trips	1.0000			−0.2967	−28.19		−0.0884	−4.17		−0.1592	−11.89
Transit Trips				1.0000			−0.1775	−8.77		−0.0848	–6.56
Bicycle Trips							1.0000			0.0791	3.39
Walk Trips										1.0000	
	Auto Accessibility			Transit Accessibility			Bicycle Accessibility			Walk Accessibility	
	Estimate	z value		Estimate	z value		Estimate	z value		Estimate	z value
*Thresholds*											
Intercept 1|2	−1.2690	−41.13		−3.5503	−60.42		−1.7417	−42.45		−0.9365	−43.36
Intercept 2|3	−0.4760	−16.03		−2.6993	−46.74		−1.0938	−27.31		−0.2938	−13.24
Intercept 3|4	0.1679	5.79		−1.7775	−31.31		−0.4989	−12.55		0.2591	11.68
Intercept 4|5	0.8520	29.01		−0.6204	−11.25		0.1817	4.61		0.9063	39.07
*Correlation Terms*											
Auto Trips	−0.0166	−1.67		–	–		–	–		–	–
Transit Trips	–	–		−0.0545	−14.21		−0.0140	−2.45		–	–
Bicycle Trips	–	–		−0.0560	−2.62		–	–		–	–
Walk Trips	–	–		–	–		–	–		–	–
Auto Accessibility	1.0000			−0.0385	−3.62		–	–		0.7734	304.72
Transit Accessibility				1.0000			0.1463	14.87		−0.0841	−8.50
Bicycle Accessibility							1.0000			–	–
Walk Accessibility										1.0000	
*Goodness of fit*	observations			AIC			BIC			likelihood (mean)	
	14,781			1774180.92			1781811.82			−886086.54	

## Data Availability

The authors do not have permission to share data.

## Footnotes

1 The authors have taken into account that aggregating integer data for accessibility to cumulative opportunities to jobs into categories can be limiting, but in this case the actual integer values for accessibility cannot be meaningfully interpreted for inferential analyses, and the sheer range of these values makes it computationally unreasonable to be used as ordered dependent categories. Therefore, we categorize modal accessibility into discrete groups which can be comparatively analysed and meaningfully interpreted.

2 A perceived problem in the latter case may be that the ordered-response model entails the estimation of N-1 threshold values that horizontally partition the underlying continuous variable to map into the observed count values, where N is the largest possible count value. But, as has been demonstrated by Meyer (1990), there is little loss of efficiency due to the estimation of a large number of thresholds in the ordered-response model structure. As long as there are a few observations in each of the N categories under consideration, it is straightforward to estimate the orderedresponse structure.
