# Supplementary material for: A joint analysis of accessibility and household trip frequencies by travel mode
Source: Transp Res Part A Policy Pract. Author manuscript; Available in PMC 2024 Mar 8. (PMC7615724; doi:10.1016/j.tra.2024.104007)
Supplement: Appendix [file EMS194538-supplement-Appendix.pdf]

## Acknowledgements

The first and third authors are supported by the Pathways to Equitable Healthy Cities grant from the Wellcome Trust [209376/Z/17/Z]. The first author is grateful to Dr. Felipe F. Dias for his help in debugging Python codes. The authors are grateful to Dr James Green for his help in editing the document. The authors are solely responsible for the contents of the paper.

## Appendix A. - A joint analysis of accessibility and household trip frequencies by travel mode

The household structure models for this paper are estimated independently for the following household structure types:
1. single adult with no children,
2. adult couple with no children,
3. single or couple with one or more children,
4. multi-adult with no children, and.
5. multi-adult with one or more children.
The results for these household structures are presented below in order of exogenous and endogenous variable effects.

**Table 1a**
Estimation results for household type Single adult with no children- Exogenous Variable Effects.

| Variable | Auto Trips | | Transit Trips | | Bicycle Trips | | Walk Trips | | Auto Access | | Transit Access | | Bicycle Access | | Walk Access | |
|---|---|---|---|---|---|---|---|---|---|---|---|---|---|---|---|---|
| | Estimate | z value | Estimate | z value | Estimate | z value | Estimate | z value | Estimate | z value | Estimate | z value | Estimate | z value | Estimate | z value |
| *(base: Age category 16–25 years)* | | | | | | | | | | | | | | | | |
| Presence in age category 26–35 years | – | – | – | – | – | – | −0.3006 | −4.01 | −0.1273 | −4.72 | −0.2613 | −3.81 | −0.1153 | −4.53 | – | – |
| Presence in age category 36–45 years | – | – | – | – | – | – | −0.4593 | −5.81 | −0.1238 | −4.35 | −0.2709 | −3.88 | – | – | – | – |
| Presence in age category 46–55 years | – | – | – | – | – | – | −0.4606 | −5.79 | – | – | −0.3034 | −4.36 | – | – | – | – |
| Presence in age category 56–65 years | – | – | – | – | – | – | −0.4837 | −6.14 | – | – | −0.2561 | −3.74 | – | – | – | – |
| Presence in age category 66 and above years | – | – | – | – | −0.1949 | −4.20 | −0.6933 | −8.45 | – | – | −0.2199 | −3.28 | – | – | – | – |
| Presence of students in the household | – | – | – | – | – | – | 0.1698 | 2.82 | 0.1061 | 2.74 | 0.1361 | 2.66 | – | – | – | – |
| Distance to CBD (km) | – | – | – | – | 0.0109 | 2.70 | −0.0492 | −11.98 | −0.0220 | −11.76 | −0.1990 | −83.37 | −0.0830 | −42.98 | 0.0168 | 9.50 |
| Population Density (Standardised value) | – | – | – | – | – | – | – | – | 0.2561 | 27.20 | 0.2268 | 24.43 | −0.0686 | −8.11 | 0.0358 | 4.03 |
| Employment Density (Standardised value) | −0.0118 | −1.41 | – | – | – | – | 0.0513 | 5.14 | −2.2480 | −124.02 | 0.4861 | 48.85 | 0.0969 | 11.52 | −0.1453 | −16.89 |
| *(base: Income less than 15,000$)* | | | | | | | | | | | | | | | | |
| Income 15,000 $-40,000$ | – | – | – | – | – | – | – | – | – | – | – | – | 0.1065 | 3.29 | – | – |
| Income 40,000$ to 60,000$ | – | – | – | – | – | – | 0.1135 | 2.71 | – | – | – | – | 0.1605 | 4.66 | – | – |
| Income 60,000$ to 100,000$ | – | – | – | – | 0.0643 | 1.44 | 0.2314 | 5.68 | −0.1588 | −6.66 | – | – | 0.1739 | 5.11 | −0.1415 | −6.28 |
| Income 100,000$ to 125,000$ | – | – | – | – | – | – | 0.2702 | 4.59 | −0.1857 | −4.69 | – | – | 0.1463 | 3.25 | −0.1936 | −4.99 |
| Income greater than 125,000 $ | – | – | – | – | – | – | 0.3531 | 5.93 | −0.2200 | −5.09 | 0.1169 | 2.99 | 0.2092 | 4.63 | −0.2194 | −5.44 |
| *(base case: trip day Friday)* | | | | | | | | | | | | | | | | |

(*continued on next page*)

**Table 1a** (*continued*)

| Variable | Auto Trips | | Transit Trips | | Bicycle Trips | | Walk Trips | | Auto Access | | Transit Access | | Bicycle Access | | Walk Access | |
|---|---|---|---|---|---|---|---|---|---|---|---|---|---|---|---|---|
| | Estimate | z value | Estimate | z value | Estimate | z value | Estimate | z value | Estimate | z value | Estimate | z value | Estimate | z value | Estimate | z value |
| Monday to Thursday as trip day | – | – | – | – | – | – | 0.0574 | 1.77 | – | – | – | – | – | – | – | – |
| *(base case: Vehicle availability per licensed adult is zero)* | | | | | | | | | | | | | | | | |
| Vehicle availability per licensed adult is one or more | – | – | – | – | – | – | −0.4836 | −15.61 | −0.0913 | −4.25 | −0.1824 | −8.65 | −0.0629 | −3.08 | −0.0392 | −1.96 |
| *(base case: Transit pass ownership zero per adult in the household)* | | | | | | | | | | | | | | | | |
| Transit pass ownership one or more per adult in the household | – | – | 0.1002 | 4.56 | 0.0933 | 2.13 | −0.0802 | −2.65 | −0.0394 | −1.89 | – | – | – | – | – | – |
| *(base case: No worker household)* | | | | | | | | | | | | | | | | |
| Single worker household | – | – | – | – | – | – | 0.1506 | 3.67 | – | – | – | – | – | – | – | – |
| *(base case: dwelling type house or townhouse)* | | | | | | | | | | | | | | | | |
| Apartment type dwelling | – | – | – | – | – | – | – | – | −0.5602 | −24.65 | – | – | – | – | −0.4927 | −22.68 |

**Table 1b**
Estimation results for household type Single adult with no children- Endogenous Variable Effects, Correlation Effects and Goodness-of-fit.

| Variable | Auto Trips | | Transit Trips | | Bicycle Trips | | Walk Trips | | Auto Access | | Transit Access | | Bicycle Access | | Walk Access | |
|---|---|---|---|---|---|---|---|---|---|---|---|---|---|---|---|---|
| | Estimate | z value | Estimate | z value | Estimate | z value | Estimate | z value | Estimate | z value | Estimate | z value | Estimate | z value | Estimate | z value |
| *(base case: Drive Access Very Low)* | | | | | | | | | | | | | | | | |
| Auto Access Low | – | – | – | – | 0.1537 | 3.37 | – | – | NA | | NA | | NA | | NA | |
| Auto Access Medium | – | – | – | – | – | – | – | – | NA | | NA | | NA | | NA | |
| Auto Access High | – | – | – | – | – | – | – | – | NA | | NA | | NA | | NA | |
| Auto Access Very High | – | – | – | – | – | – | – | – | NA | | NA | | NA | | NA | |
| *(base case: Transit Access Very Low)* | | | | | | | | | | | | | | | | |
| Transit Access Low | – | – | – | – | 0.1237 | 2.14 | – | – | NA | | NA | | NA | | NA | |
| Transit Access Medium | – | – | – | – | – | – | – | – | NA | | NA | | NA | | NA | |
| Transit Access High | – | – | – | – | 0.1268 | 2.56 | 0.1246 | 2.60 | NA | | NA | | NA | | NA | |
| Transit Access Very High | – | – | – | – | – | – | 0.3114576 | 5.66 | NA | | NA | | NA | | NA | |
| *(base case: Bicycle Access Very Low)* | | | | | | | | | | | | | | | | |
| Bicycle Access Low | – | – | – | – | – | – | – | – | NA | | NA | | NA | | NA | |
| Bicycle Access Medium | – | – | – | – | – | – | – | – | NA | | NA | | NA | | NA | |
| Bicycle Access High | – | – | – | – | – | – | −0.0723 | −1.92 | NA | | NA | | NA | | NA | |
| Bicycle Access Very High | – | – | – | – | – | – | −0.0572 | −1.56 | NA | | NA | | NA | | NA | |
| *(base case: Walk Access Very Low)* | | | | | | | | | | | | | | | | |
| Walk Access Low | – | – | – | – | – | – | 0.2028 | 6.34 | NA | | NA | | NA | | NA | |
| Walk Access Medium | – | – | – | – | – | – | – | – | NA | | NA | | NA | | NA | |
| Walk Access High | – | – | – | – | – | – | – | – | NA | | NA | | NA | | NA | |
| Walk Access Very High | – | – | – | – | – | – | – | – | NA | | NA | | NA | | NA | |
| *Thresholds* | | | | | | | | | | | | | | | | |

(*continued on next page*)

**Table 1b** (*continued*)

| Variable | Auto Trips | | Transit Trips | | Bicycle Trips | | Walk Trips | | Auto Access | | Transit Access | | Bicycle Access | | Walk Access | |
|---|---|---|---|---|---|---|---|---|---|---|---|---|---|---|---|---|
| | Estimate | z value | Estimate | z value | Estimate | z value | Estimate | z value | Estimate | z value | Estimate | z value | Estimate | z value | Estimate | z value |
| Intercept 1\|2 | 0.1603 | 14.77 | 0.5124 | 38.24 | 1.9825 | 35.99 | 0.4503 | 4.37 | −1.5844 | −45.59 | −3.7638 | −51.97 | −1.6796 | −49.12 | −1.2224 | −41.77 |
| Intercept 2\|3 | 0.8865 | 70.80 | 0.6314 | 46.27 | 1.9998 | 36.13 | 0.7594 | 7.37 | −0.6136 | −18.28 | −2.9544 | −41.15 | −1.0763 | −32.58 | −0.4501 | −15.20 |
| Intercept 3\|4 | 1.6155 | 90.37 | 1.6120 | 85.11 | 2.4181 | 39.99 | 1.6307 | 15.51 | 0.0666 | 2.02 | −2.0870 | −29.58 | −0.4879 | −14.99 | 0.1482 | 5.02 |
| Intercept 4\|5 | 2.4113 | 68.48 | 2.5996 | 61.98 | 2.9227 | 36.67 | 2.4940 | 21.65 | 0.7600 | 22.68 | −0.8620 | −12.68 | 0.1963 | 6.10 | 0.7979 | 26.19 |
| *Correlation Terms* | | | | | | | | | | | | | | | | |
| Auto Trips | 1.0000 | | −0.6048 | −74.13 | −0.3794 | −17.30 | −0.0839 | −5.77 | −0.0439 | −4.04 | – | – | – | – | −0.0407 | −3.93 |
| Transit Trips | | | 1.0000 | | −0.3648 | −14.06 | −0.1243 | −8.30 | −0.0514 | −4.43 | – | – | – | – | −0.0296 | −2.63 |
| Bicycle Trips | | | | | 1.0000 | | −0.2215 | −10.73 | −0.0483 | −2.30 | – | – | – | – | −0.0518 | −2.64 |
| Walk Trips | | | | | | | 1.0000 | | – | – | −0.0233 | −2.17 | – | – | – | – |
| Drive Access | | | | | | | | | 1.0000 | | – | – | – | – | 0.7094 | 223.94 |
| Transit Access | | | | | | | | | | | 1.0000 | | 0.1668 | 16.25 | −0.0729 | −7.17 |
| Bicycle Access | | | | | | | | | | | | | 1.0000 | | – | – |
| Walk Access | | | | | | | | | | | | | | | 1.0000 | |
| *Goodness of fit* | Link | | Function threshold | | Observations | | No. of dimensions | | Likelihood (mean) | | AIC | | BIC | | Function evals. | |
| | Multivariate probit | | flexible | | 13,613 | | 8 | | −775220.38 | | 1551927.59 | | 1557517.20 | | 26,176 | |

**Table 2a**
Estimation results for household type Couple adults with no children - Exogenous Variable Effects.

| Variable | Auto Trips | | Transit Trips | | Bicycle Trips | | Walk Trips | | Auto Access | | Transit Access | | Bicycle Access | | Walk Access | |
|---|---|---|---|---|---|---|---|---|---|---|---|---|---|---|---|---|
| | Estimate | z value | Estimate | z value | Estimate | z value | Estimate | z value | Estimate | z value | Estimate | z value | Estimate | z value | Estimate | z value |
| *(base: Age category 16–25 years)* | | | | | | | | | | | | | | | | |
| Presence in age category 26–35 years | −0.1034 | −3.86 | 0.2355 | 7.80 | – | – | – | – | – | – | – | – | −0.1074 | −5.25 | – | – |
| Presence in age category 36–45 years | −0.0531 | −1.93 | 0.1625 | 5.43 | – | – | – | – | – | – | – | – | −0.0886 | −3.71 | – | – |
| Presence in age category 46–55 years | −0.0788 | −3.14 | 0.1501 | 5.16 | – | – | – | – | – | – | – | – | – | – | – | – |
| Presence in age category 56–65 years | 0.1172 | 4.34 | – | – | – | – | – | – | – | – | – | – | – | – | – | – |
| Presence in age category 66 and above years | – | – | – | – | −0.4601 | −7.62 | −0.1022 | −2.62 | – | – | – | – | – | – | – | – |
| Presence of students in the household | – | – | 0.2819 | 8.37 | 0.3018 | 5.58 | 0.1721 | 4.04 | – | – | – | – | – | – | – | – |
| Distance to CBD (km) | 0.0113 | 4.88 | 0.0118 | 4.07 | −0.0931 | −21.47 | −0.0705 | −22.97 | −0.0133 | −7.91 | −0.1852 | −80.81 | −0.0838 | −47.01 | 0.0207 | 13.34 |
| Population Density (Standardised value) | −0.0291 | −2.77 | – | – | −0.0973 | −4.49 | 0.0450 | 3.42 | 0.2365 | 25.66 | 0.2489 | 24.69 | – | – | – | – |
| Employment Density (Standardised value) | −0.0208 | −2.18 | −0.0526 | −5.06 | −0.1252 | −6.13 | 0.0683 | 6.23 | −2.1572 | −112.71 | 0.4428 | 44.02 | 0.1059 | 13.24 | −0.1416 | −15.58 |
| *(base: Income less than 15,000$)* | | | | | | | | | | | | | | | | |
| Income 15,000 $-40,000$ | – | – | 0.0933 | 1.56 | – | – | – | – | 0.1358 | 5.51 | −0.1255 | −2.31 | – | – | 0.0970 | 4.08 |
| Income 40,000$ to 60,000$ | 0.1997 | 6.38 | 0.1084 | 1.76 | – | – | – | – | – | – | −0.1140 | −2.07 | 0.1042 | 3.39 | – | – |
| Income 60,000$ to 100,000$ | 0.3342 | 11.29 | 0.2287 | 3.85 | – | – | – | – | – | – | −0.1124 | −2.11 | 0.1244 | 4.49 | – | – |
| Income 100,000$ to 125,000$ | 0.4032 | 11.51 | 0.3286 | 5.18 | – | – | – | – | – | – | −0.1576 | −2.81 | 0.1408 | 4.36 | – | – |
| Income greater than 125,000$ | 0.4907 | 15.35 | 0.3162 | 5.08 | – | – | – | – | – | – | −0.0951 | −1.76 | 0.2282 | 8.09 | – | – |
| *(base: fraction of male adults in the household)* | | | | | | | | | | | | | | | | |

(*continued on next page*)

**Table 2a** (*continued*)

| Variable | Auto Trips | | Transit Trips | | Bicycle Trips | | Walk Trips | | Auto Access | | Transit Access | | Bicycle Access | | Walk Access | |
|---|---|---|---|---|---|---|---|---|---|---|---|---|---|---|---|---|
| | Estimate | z value | Estimate | z value | Estimate | z value | Estimate | z value | Estimate | z value | Estimate | z value | Estimate | z value | Estimate | z value |
| fraction of female adults in the household | – | – | 0.1909 | 3.94 | −0.2627 | −2.94 | – | – | – | – | – | – | 0.1029 | 2.33 | – | – |
| *(base case: Vehicle availability per licensed adult is zero)* | | | | | | | | | | | | | | | | |
| Vehicle availability per licensed adult is between zero and one | 1.3931 | 42.01 | −0.5656 | −19.43 | −0.4609 | −10.46 | −0.2780 | −8.47 | −0.0866 | −3.57 | −0.0716 | −2.45 | – | – | – | – |
| Vehicle availability per licensed adults as one or more | 1.6123 | 47.18 | −1.0136 | −33.66 | −0.9081 | −17.11 | −0.5488 | −15.76 | −0.0794 | −3.35 | −0.1735 | −5.95 | – | – | – | – |
| *(base case: Transit pass ownership less than one per adult in the household)* | | | | | | | | | | | | | | | | |
| Transit pass ownership one or more per adult in the household | −0.3582 | −12.79 | 0.7115 | 26.88 | −0.2557 | −5.86 | – | – | – | – | – | – | – | – | – | – |
| *(base case: No worker household)* | | | | | | | | | | | | | | | | |
| Single worker household | – | – | 0.4793 | 14.27 | – | – | 0.2339 | 5.44 | – | – | 0.0531 | 2.39 | – | – | – | – |
| Multi-worker household | 0.1170 | 5.05 | 0.5985 | 18.12 | 0.2250 | 4.60 | 0.3034 | 6.90 | – | – | – | – | – | – | – | – |
| *(base case: dwelling type house or townhouse)* | | | | | | | | | | | | | | | | |
| Apartment type dwelling | −0.1020 | −4.72 | 0.1426 | 5.83 | −0.3216 | −7.21 | – | – | −0.6051 | −28.92 | – | – | −0.1321 | −6.84 | −0.4568 | −24.07 |

**Table 2b**

Estimation results for household type Couple adults with no children - Endogenous Variable Effects, Correlation Effects and Goodness-of-fit.

| Variable | Auto Trips | | Transit Trips | | Bicycle Trips | | Walk Trips | | Auto Access | | Transit Access | | Bicycle Access | | Walk Access | |
|---|---|---|---|---|---|---|---|---|---|---|---|---|---|---|---|---|
| | Estimate | z value | Estimate | z value | Estimate | z value | Estimate | z value | Estimate | z value | Estimate | z value | Estimate | z value | Estimate | z value |
| *(base case: Drive Access Very Low)* | | | | | | | | | | | | | | | | |
| Auto Access Low | – | – | 0.0539 | 1.95 | – | – | – | – | NA | | NA | | NA | | NA | |
| Auto Access Medium | – | – | 0.0521 | 1.87 | – | – | – | – | NA | | NA | | NA | | NA | |
| Auto Access High | – | – | – | – | – | – | – | – | NA | | NA | | NA | | NA | |
| Auto Access Very High | – | – | – | – | – | – | – | – | NA | | NA | | NA | | NA | |
| *(base case: Transit Access Very Low)* | | | | | | | | | | | | | | | | |
| Transit Access Low | −0.0510 | −1.99 | 0.0967 | 2.60 | – | – | – | – | NA | | NA | | NA | | NA | |
| Transit Access Medium | – | – | 0.1826 | 4.91 | – | – | −0.1469 | −3.71 | NA | | NA | | NA | | NA | |
| Transit Access High | −0.0832 | −2.80 | 0.1913 | 4.63 | – | – | – | – | NA | | NA | | NA | | NA | |
| Transit Access Very High | −0.2576 | −7.14 | 0.1081 | 2.32 | – | – | 0.1610732 | 4.35 | NA | | NA | | NA | | NA | |
| *(base case: Bicycle Access Very Low)* | | | | | | | | | | | | | | | | |
| Bicycle Access Low | – | – | −0.0682 | −2.23 | – | – | – | – | NA | | NA | | NA | | NA | |
| Bicycle Access Medium | – | – | – | – | – | – | – | – | NA | | NA | | NA | | NA | |
| Bicycle Access High | – | – | 0.0742 | 2.56 | – | – | – | – | NA | | NA | | NA | | NA | |
| Bicycle Access Very High | – | – | 0.1351 | 4.56 | – | – | – | – | NA | | NA | | NA | | NA | |
| *(base case: Walk Access Very Low)* | | | | | | | | | | | | | | | | |

(*continued on next page*)

**Table 2b** (*continued*)

| Variable | Auto Trips Estimate | z value | Transit Trips Estimate | z value | Bicycle Trips Estimate | z value | Walk Trips Estimate | z value | Auto Access Estimate | z value | Transit Access Estimate | z value | Bicycle Access Estimate | z value | Walk Access Estimate | z value |
|---|---|---|---|---|---|---|---|---|---|---|---|---|---|---|---|---|
| Walk Access Low | – | – | −0.1940 | −6.79 | −0.1271 | −2.54 | 0.2120 | 6.24 | NA | | NA | | NA | | NA | |
| Walk Access Medium | −0.0466 | −1.99 | – | – | – | – | 0.0368 | 1.06 | NA | | NA | | NA | | NA | |
| Walk Access High | – | – | – | – | – | – | – | – | NA | | NA | | NA | | NA | |
| Walk Access Very High | – | – | – | – | – | – | – | – | NA | | NA | | NA | | NA | |
| *Thresholds* | | | | | | | | | | | | | | | | |
| Intercept 1\|2 | 0.9848 | 19.53 | 1.0427 | 12.29 | 0.0686 | 0.85 | 0.4225 | 7.17 | −1.2690 | −41.13 | −3.5503 | −60.42 | −1.7417 | −42.45 | −0.9365 | −43.36 |
| Intercept 2\|3 | 1.7602 | 34.12 | 1.1854 | 13.97 | 0.1098 | 1.35 | 0.6582 | 11.10 | −0.4760 | −16.03 | −2.6993 | −46.74 | −1.0938 | −27.31 | −0.2938 | −13.24 |
| Intercept 3\|4 | 2.4563 | 46.93 | 2.1584 | 25.33 | 0.6315 | 7.59 | 1.3287 | 22.46 | 0.1679 | 5.79 | −1.7775 | −31.31 | −0.4989 | −12.55 | 0.2591 | 11.68 |
| Intercept 4\|5 | 3.1817 | 59.84 | 3.1570 | 35.97 | 1.1619 | 13.20 | 2.1287 | 33.62 | 0.8520 | 29.01 | −0.6204 | −11.25 | 0.1817 | 4.61 | 0.9063 | 39.07 |
| *Correlation Terms* | | | | | | | | | | | | | | | | |
| Auto Trips | 1.0000 | | −0.2967 | −28.19 | −0.0884 | −4.17 | −0.1592 | −11.89 | −0.0166 | −1.67 | – | – | – | – | – | – |
| Transit Trips | | | 1.0000 | | −0.1775 | −8.77 | −0.0848 | −6.56 | – | – | −0.0545 | −14.21 | −0.0140 | −2.45 | – | – |
| Bicycle Trips | | | | | 1.0000 | | 0.0791 | 3.39 | – | – | −0.0560 | −2.62 | – | – | – | – |
| Walk Trips | | | | | | | 1.0000 | | – | – | – | – | – | – | – | – |
| Drive Access | | | | | | | | | 1.0000 | | −0.0385 | −3.62 | – | – | 0.7734 | 304.72 |
| Transit Access | | | | | | | | | | | 1.0000 | | 0.1463 | 14.87 | −0.0841 | −8.50 |
| Bicycle Access | | | | | | | | | | | | | 1.0000 | | – | – |
| Walk Access | | | | | | | | | | | | | | | 1.0000 | |
| *Goodness of fit* | Link | | Function threshold | | Observations | | No. of dimensions | | Likelihood (mean) | | AIC | | BIC | | Function evals. | |
| | Multivariate probit | | flexible | | 14,781 | | 8 | | −886086.54 | | 1774180.92 | | 1781811.82 | | 6195 | |

**Table 3a**

Estimation results for household type Single or Couple adults with one or more children- Exogenous Variable Effects.

| Variable | Auto Trips Estimate | z value | Transit Trips Estimate | z value | Bicycle Trips Estimate | z value | Walk Trips Estimate | z value | Auto Access Estimate | z value | Transit Access Estimate | z value | Bicycle Access Estimate | z value | Walk Access Estimate | z value |
|---|---|---|---|---|---|---|---|---|---|---|---|---|---|---|---|---|
| *(base: Presence in age category 0–15 years)* | | | | | | | | | | | | | | | | |
| Presence in age category 16–25 years | – | – | 0.5114 | 6.46 | – | – | – | – | −0.1434 | −2.00 | −0.1589 | −1.80 | – | – | – | – |
| Presence in age category 26–35 years | – | – | −0.0789 | −2.03 | – | – | −0.1552 | −3.36 | – | – | – | – | – | – | 0.0557 | 1.86 |
| Presence in age category 36–45 years | – | – | – | – | – | – | 0.0956 | 2.37 | – | – | – | – | – | – | – | – |
| Presence in age category 46–55 years | 0.1370 | 4.38 | 0.2556 | 7.43 | – | – | 0.1622 | 3.82 | – | – | 0.0598 | 1.96 | – | – | – | – |
| Presence in age category 56–65 years | – | – | – | – | – | – | – | – | – | – | – | – | – | – | – | – |
| Presence in age category 66 and above years | – | – | – | – | – | – | 0.2506 | 2.01 | – | – | – | – | – | – | – | – |
| Presence of students in the household | 0.2023 | 6.07 | 0.3316 | 8.63 | – | – | 0.3321 | 8.16 | – | – | – | – | – | – | 0.0607 | 2.15 |
| Distance to CBD (km) | 0.0114 | 3.26 | 0.0092 | 2.54 | −0.1073 | −17.86 | −0.0317 | −9.47 | −0.0305 | −12.10 | −0.1957 | −51.79 | −0.0797 | −28.14 | 0.0144 | 5.73 |
| Population Density (Standardised value) | −0.0488 | −2.50 | – | – | −0.1214 | −3.07 | – | – | 0.2774 | 17.39 | 0.2278 | 12.66 | 0.0422 | 2.81 | 0.0517 | 2.92 |
| Employment Density (Standardised value) | −0.0566 | −2.65 | −0.0620 | −3.39 | – | – | 0.0579 | 2.97 | −4.5830 | −69.49 | 0.5446 | 30.97 | 0.0812 | 4.67 | −0.1869 | −13.21 |
| *(base: Income less than 15,000$)* | | | | | | | | | | | | | | | | |
| Income 15,000 $-40,000$ | – | – | – | – | – | – | 0.1261 | 2.55 | – | – | – | – | – | – | – | – |

(*continued on next page*)

**Table 3a** (*continued*)

| Variable | Auto Trips | | Transit Trips | | Bicycle Trips | | Walk Trips | | Auto Access | | Transit Access | | Bicycle Access | | Walk Access | |
|---|---|---|---|---|---|---|---|---|---|---|---|---|---|---|---|---|
| | Estimate | z value | Estimate | z value | Estimate | z value | Estimate | z value | Estimate | z value | Estimate | z value | Estimate | z value | Estimate | z value |
| Income 40,000$ to 60,000$ | – | – | 0.0897 | 1.58 | – | – | – | – | – | – | −0.1369 | −2.48 | – | – | – | – |
| Income 60,000$ to 100,000$ | – | – | 0.1633 | 3.11 | 0.1132 | 1.71 | – | – | – | – | −0.0852 | −1.73 | 0.1521 | 3.72 | – | – |
| Income 100,000$ to 125,000$ | 0.1755 | 3.90 | 0.2209 | 3.71 | – | – | – | – | 0.0622 | 1.73 | −0.1863 | −3.36 | 0.2569 | 5.25 | – | – |
| Income greater than 125,000$ | 0.2892 | 8.06 | 0.1297 | 2.42 | – | – | 0.1024 | 2.80 | – | – | −0.1798 | −3.68 | 0.3555 | 8.92 | – | – |
| *(base case: Vehicle availability per licensed adult is zero)* | | | | | | | | | | | | | | | | |
| Vehicle availability per licensed adult is between zero and one | 1.7215 | 28.18 | −0.5539 | −10.77 | −0.2064 | −2.62 | −0.2062 | −4.00 | – | – | – | – | −0.1083 | −3.89 | – | – |
| Vehicle availability per licensed adults as one or more | 2.0362 | 33.23 | −1.0408 | −20.21 | −0.7252 | −8.85 | −0.5379 | −10.32 | – | – | −0.2032 | −6.93 | – | – | – | – |
| *(base case: Transit pass ownership less than one per adult in the household)* | | | | | | | | | | | | | | | | |
| Transit pass ownership one or more per adult in the household | −0.4767 | −12.10 | 0.7720 | 18.89 | – | – | – | – | – | – | −0.0751 | −1.85 | −0.1077 | −2.90 | – | – |
| *(base case: No worker household)* | | | | | | | | | | | | | | | | |
| Single worker household | – | – | 0.4550 | 6.72 | – | – | – | – | – | – | – | – | – | – | – | – |
| Multi-worker household | 0.1793 | 6.04 | 0.6902 | 9.91 | – | – | – | – | – | – | – | – | – | – | – | – |
| *(base case: dwelling type house or townhouse)* | | | | | | | | | | | | | | | | |
| Apartment type dwelling | −0.1382 | −3.75 | 0.1231 | 3.38 | −0.5461 | −7.08 | – | – | −0.3682 | −10.68 | −0.0758 | −2.06 | −0.2031 | −5.90 | −0.3865 | −10.95 |

**Table 3b**
Estimation results for household type Single or Couple adults with one or more children - Endogenous Variable Effects, Correlation Effects and Goodness-of-fit.

| Variable | Auto Trips | | Transit Trips | | Bicycle Trips | | Walk Trips | | Auto Access | | Transit Access | | Bicycle Access | | Walk Access | |
|---|---|---|---|---|---|---|---|---|---|---|---|---|---|---|---|---|
| | Estimate | z value | Estimate | z value | Estimate | z value | Estimate | z value | Estimate | z value | Estimate | z value | Estimate | z value | Estimate | z value |
| *(base case: Drive Access Very Low)* | | | | | | | | | | | | | | | | |
| Auto Access Low | – | – | – | – | – | – | – | – | NA | | NA | | NA | | NA | |
| Auto Access Medium | – | – | – | – | – | – | 0.1205 | 3.22 | NA | | NA | | NA | | NA | |
| Auto Access High | 0.1272 | 2.88 | – | – | – | – | – | – | NA | | NA | | NA | | NA | |
| Auto Access Very High | – | – | – | – | – | – | – | – | NA | | NA | | NA | | NA | |
| *(base case: Transit Access Very Low)* | | | | | | | | | | | | | | | | |
| Transit Access Low | – | – | – | – | – | – | – | – | NA | | NA | | NA | | NA | |
| Transit Access Medium | – | – | – | – | – | – | – | – | NA | | NA | | NA | | NA | |
| Transit Access High | −0.1847 | −4.48 | 0.1298 | 3.02 | – | – | – | – | NA | | NA | | NA | | NA | |
| Transit Access Very High | −0.2574 | −5.03 | 0.1336 | 2.54 | 0.1724 | 3.57 | – | – | NA | | NA | | NA | | NA | |
| *(base case: Bicycle Access Very Low)* | | | | | | | | | | | | | | | | |
| Bicycle Access Low | – | – | – | – | – | – | – | – | NA | | NA | | NA | | NA | |
| Bicycle Access Medium | – | – | – | – | – | – | – | – | NA | | NA | | NA | | NA | |
| Bicycle Access High | – | – | – | – | – | – | – | – | NA | | NA | | NA | | NA | |
| Bicycle Access Very High | 0.0662 | 1.79 | – | – | – | – | 0.1098 | 2.72 | NA | | NA | | NA | | NA | |
| *(base case: Walk Access Very Low)* | | | | | | | | | | | | | | | | |

(*continued on next page*)

**Table 3b** (*continued*)

| Variable | Auto Trips | | Transit Trips | | Bicycle Trips | | Walk Trips | | Auto Access | | Transit Access | | Bicycle Access | | Walk Access | |
|---|---|---|---|---|---|---|---|---|---|---|---|---|---|---|---|---|
| | Estimate | z value | Estimate | z value | Estimate | z value | Estimate | z value | Estimate | z value | Estimate | z value | Estimate | z value | Estimate | z value |
| Walk Access Low | – | – | – | – | −0.3357 | −3.85 | – | – | NA | | NA | | NA | | NA | |
| Walk Access Medium | – | – | – | – | −0.1736 | −2.30 | – | – | NA | | NA | | NA | | NA | |
| Walk Access High | −0.1187 | −2.68 | – | – | – | – | – | – | NA | | NA | | NA | | NA | |
| Walk Access Very High | – | – | – | – | – | – | – | – | NA | | NA | | NA | | NA | |
| *Thresholds* | | | | | | | | | | | | | | | | |
| Intercept 1\|2 | 1.1966 | 14.31 | 0.5299 | 5.55 | – | – | 0.2109 | 2.70 | −0.7790 | −21.16 | −3.7166 | −51.01 | −1.7463 | −33.91 | −0.7575 | −20.94 |
| Intercept 2\|3 | 2.0196 | 24.07 | 0.6904 | 7.22 | – | – | 0.4059 | 5.18 | −0.1003 | −2.74 | −2.8147 | −40.56 | −1.0691 | −21.25 | −0.3705 | −10.16 |
| Intercept 3\|4 | 2.6194 | 31.01 | 1.6333 | 16.85 | 0.5940 | 6.91 | 0.9880 | 12.52 | 0.5966 | 16.60 | −1.8248 | −27.73 | −0.5061 | −10.17 | 0.1431 | 3.94 |
| Intercept 4\|5 | 3.2851 | 38.22 | 2.5227 | 25.40 | 1.1627 | 12.14 | 1.6765 | 20.61 | 1.3850 | 37.22 | −0.7112 | −11.19 | 0.1950 | 3.94 | 0.8383 | 22.40 |
| *Correlation Terms* | | | | | | | | | | | | | | | | |
| Auto Trips | 1.0000 | | −0.2507 | −17.92 | −0.2227 | −8.65 | −0.1249 | −7.79 | – | – | – | – | 0.0192 | 1.68 | – | – |
| Transit Trips | | | 1.0000 | | −0.2798 | −12.05 | −0.1081 | −6.33 | – | – | – | – | – | – | – | – |
| Bicycle Trips | | | | | 1.0000 | | – | – | – | – | – | – | – | – | – | – |
| Walk Trips | | | | | | | 1.0000 | | – | – | – | – | – | – | – | – |
| Drive Access | | | | | | | | | 1.0000 | | – | – | – | – | 0.7725 | 168.46 |
| Transit Access | | | | | | | | | | | 1.0000 | | 0.0915 | 6.27 | −0.1282 | −8.77 |
| Bicycle Access | | | | | | | | | | | | | 1.0000 | | – | – |
| Walk Access | | | | | | | | | | | | | | | 1.0000 | |
| *Goodness of fit* | Link | | Function threshold | | Observations | | No. of dimensions | | Likelihood (mean) | | AIC | | BIC | | Function evals. | |
| | Multivariate probit | | flexible | | 6526 | | 8 | | −432115.50 | | 866000.98 | | 872004.35 | | 27,278 | |

**Table 4a**

Estimation results for household type Multi adults with no children- Exogenous Variable Effects.

| Variable | Auto Trips | | Transit Trips | | Bicycle Trips | | Walk Trips | | Auto Access | | Transit Access | | Bicycle Access | | Walk Access | |
|---|---|---|---|---|---|---|---|---|---|---|---|---|---|---|---|---|
| | Estimate | z value | Estimate | z value | Estimate | z value | Estimate | z value | Estimate | z value | Estimate | z value | Estimate | z value | Estimate | z value |
| *(base: Presence in age category 16–25 years)* | | | | | | | | | | | | | | | | |
| Presence in age category 26–35 years | – | – | – | – | – | – | – | – | – | – | – | – | – | – | – | – |
| Presence in age category 36–45 years | – | – | – | – | – | – | 0.1169 | 2.04 | – | – | – | – | – | – | 0.0669 | 1.94 |
| Presence in age category 46–55 years | – | – | – | – | −0.1830 | −2.40 | – | – | – | – | – | – | – | – | – | – |
| Presence in age category 56–65 years | 0.0722 | 2.44 | −0.2112 | −2.56 | – | – | −0.1558 | −3.57 | – | – | – | – | – | – | – | – |
| Presence in age category 66 and above years | – | – | −0.0949 | −2.50 | −0.3613 | −4.03 | −0.2219 | −4.45 | – | – | 0.0923 | 2.83 | – | – | – | – |
| Presence of students in the household | 0.0861 | 2.29 | 0.4667 | 12.13 | – | – | 0.2818 | 6.33 | – | – | – | – | – | – | – | – |
| Distance to CBD (km) | 0.0101 | 2.65 | −0.0113 | −3.78 | −0.0652 | −7.87 | −0.0311 | −6.45 | −0.0308 | −11.31 | −0.1785 | −46.15 | −0.0946 | −34.32 | 0.0114 | 4.42 |
| Population Density (Standardised value) | – | – | – | – | −0.1818 | −2.43 | – | – | 0.2894 | 17.12 | 0.2676 | 14.01 | – | – | 0.0327 | 1.87 |
| Employment Density (Standardised value) | – | – | −0.0785 | −3.20 | – | – | 0.1124 | 3.64 | −4.5559 | −58.60 | 0.7652 | 28.06 | 0.1038 | 4.72 | −0.2364 | −14.33 |
| *(base: Income less than 15,000$)* | | | | | | | | | | | | | | | | |

(*continued on next page*)

**Table 4a** (*continued*)

| Variable | Auto Trips | | Transit Trips | | Bicycle Trips | | Walk Trips | | Auto Access | | Transit Access | | Bicycle Access | | Walk Access | |
|---|---|---|---|---|---|---|---|---|---|---|---|---|---|---|---|---|
| | Estimate | z value | Estimate | z value | Estimate | z value | Estimate | z value | Estimate | z value | Estimate | z value | Estimate | z value | Estimate | z value |
| Income 15,000 $-40,000$ | – | – | – | – | – | – | – | – | – | – | – | – | – | – | – | – |
| Income 40,000$ to 60,000$ | 0.1159 | 2.27 | – | – | – | – | – | – | – | – | −0.0867 | −2.06 | – | – | – | – |
| Income 60,000$ to 100,000$ | 0.2349 | 4.96 | 0.1074 | 2.88 | 0.2481 | 2.28 | – | – | – | – | – | – | – | – | – | – |
| Income 100,000$ to 125,000$ | 0.3655 | 6.63 | – | – | 0.2657 | 2.02 | – | – | – | – | – | – | – | – | – | – |
| Income greater than 125,000$ | 0.5499 | 10.92 | 0.0695 | 1.81 | 0.4374 | 3.92 | – | – | – | – | – | – | 0.2238 | 6.4497 | – | – |
| *(base: fraction of male adults in the household)* | | | | | | | | | | | | | | | | |
| fraction of female adults in the household | – | – | 0.2253 | 3.03 | −0.5511 | −3.03 | – | – | – | – | 0.1694 | 2.16 | – | – | – | – |
| *(base case: Vehicle availability per licensed adult is zero)* | | | | | | | | | | | | | | | | |
| Vehicle availability per licensed adult is between zero and one | 1.7489 | 24.58 | −0.7648 | −13.26 | −0.4690 | −4.36 | −0.3625 | −5.47 | – | – | – | – | – | – | – | – |
| Vehicle availability per licensed adults as one or more | 1.8161 | 24.75 | −1.0752 | −17.90 | −0.8886 | −7.08 | −0.5787 | −8.03 | – | – | −0.0699 | −2.22 | – | – | – | – |
| *(base case: Transit pass ownership less than one per adult in the household)* | | | | | | | | | | | | | | | | |
| Transit pass ownership one or more per adult in the household | −0.5758 | −8.14 | 0.7991 | 11.79 | – | – | −0.3427 | −3.69 | – | – | – | – | – | – | – | – |
| *(base case: No worker household)* | | | | | | | | | | | | | | | | |
| Single worker household | – | – | 0.4041 | 5.61 | – | – | – | – | – | – | – | – | – | – | – | – |
| Multi-worker household | 0.1823 | 5.25 | 0.7590 | 11.15 | – | – | 0.1873 | 3.71 | −0.0591 | −2.13 | – | – | −0.0408 | −1.24 | – | – |
| *(base case: dwelling type house or townhouse)* | | | | | | | | | | | | | | | | |
| Apartment type dwelling | −0.1850 | −5.01 | 0.1391 | 3.70 | −0.3506 | −3.05 | – | – | −0.3361 | −7.82 | −0.0778 | −1.96 | −0.2119 | −6.27 | −0.3266 | −7.75 |

**Table 4b**

Estimation results for household type Multi adults with no children - Endogenous Variable Effects, Correlation Effects and Goodness-of-fit.

| Variable | Auto Trips | | Transit Trips | | Bicycle Trips | | Walk Trips | | Auto Access | | Transit Access | | Bicycle Access | | Walk Access | |
|---|---|---|---|---|---|---|---|---|---|---|---|---|---|---|---|---|
| | Estimate | z value | Estimate | z value | Estimate | z value | Estimate | z value | Estimate | z value | Estimate | z value | Estimate | z value | Estimate | z value |
| *(base case: Drive Access Very Low)* | | | | | | | | | | | | | | | | |
| Auto Access Low | – | – | – | – | – | – | 0.2029 | 3.04 | NA | | NA | | NA | | NA | |
| Auto Access Medium | – | – | – | – | – | – | 0.1415 | 2.07 | NA | | NA | | NA | | NA | |
| Auto Access High | – | – | – | – | – | – | – | – | NA | | NA | | NA | | NA | |
| Auto Access Very High | – | – | 0.0619 | 1.76 | – | – | – | – | NA | | NA | | NA | | NA | |
| *(base case: Transit Access Very Low)* | | | | | | | | | | | | | | | | |
| Transit Access Low | −0.1317 | −3.29 | 0.0919 | 2.39 | – | – | – | – | NA | | NA | | NA | | NA | |
| Transit Access Medium | −0.1821 | −4.11 | 0.1199 | 2.95 | – | – | – | – | NA | | NA | | NA | | NA | |
| Transit Access High | −0.2937 | −4.72 | 0.1090 | 2.08 | – | – | 0.1719 | 2.48 | NA | | NA | | NA | | NA | |
| Transit Access Very High | −0.4822 | −6.49 | | | 0.2759 | 2.62 | 0.4256493 | 5.12 | NA | | NA | | NA | | NA | |

(*continued on next page*)

**Table 4b** (*continued*)

| Variable | Auto Trips | | Transit Trips | | Bicycle Trips | | Walk Trips | | Auto Access | | Transit Access | | Bicycle Access | | Walk Access | |
|---|---|---|---|---|---|---|---|---|---|---|---|---|---|---|---|---|
| | Estimate | z value | Estimate | z value | Estimate | z value | Estimate | z value | Estimate | z value | Estimate | z value | Estimate | z value | Estimate | z value |
| *(base case: Bicycle Access Very Low)* | | | | | | | | | | | | | | | | |
| Bicycle Access Low | – | – | – | – | – | – | – | – | NA | | NA | | NA | | NA | |
| Bicycle Access Medium | – | – | – | – | −0.1991 | −1.93 | – | – | NA | | NA | | NA | | NA | |
| Bicycle Access High | – | – | – | – | – | – | 0.1314 | 2.59 | NA | | NA | | NA | | NA | |
| Bicycle Access Very High | – | – | – | – | – | – | – | – | NA | | NA | | NA | | NA | |
| *(base case: Walk Access Very Low)* | | | | | | | | | | | | | | | | |
| Walk Access Low | −0.1627 | −3.51 | – | – | – | – | – | – | NA | | NA | | NA | | NA | |
| Walk Access Medium | – | – | – | – | – | – | −0.1617 | −2.32 | NA | | NA | | NA | | NA | |
| Walk Access High | – | – | – | – | – | – | – | – | NA | | NA | | NA | | NA | |
| Walk Access Very High | – | – | – | – | – | – | – | – | NA | | NA | | NA | | NA | |
| *Thresholds* | | | | | | | | | | | | | | | | |
| Intercept 1\|2 | 1.0069 | 9.61 | – | – | – | – | 0.4553 | 4.22 | −0.6664 | −13.33 | −3.4865 | −48.59 | −2.0532 | −41.63 | −0.7619 | −19.18 |
| Intercept 2\|3 | 1.8277 | 17.19 | – | – | – | – | 0.6905 | 6.41 | −0.1113 | −2.21 | −2.4184 | −35.92 | −1.3183 | −27.71 | −0.3997 | −9.98 |
| Intercept 3\|4 | 2.4441 | 22.81 | 0.9750 | 9.76 | 0.8981 | 4.39 | 1.4981 | 13.55 | 0.4927 | 9.97 | −1.3178 | −21.13 | −0.7082 | −15.22 | 0.1135 | 2.85 |
| Intercept 4\|5 | 3.0969 | 28.64 | 1.8175 | 17.89 | 1.4040 | 6.96 | 2.0735 | 17.93 | 1.1977 | 23.84 | −0.2670 | −4.42 | 0.0822 | 1.80 | 0.7322 | 17.92 |
| *Correlation Terms* | | | | | | | | | | | | | | | | |
| Auto Trips | 1.0000 | | −0.2817 | −19.77 | −0.1971 | −5.94 | −0.0724 | −3.34 | – | – | – | – | – | – | – | – |
| Transit Trips | | | 1.0000 | | −0.2030 | −6.32 | −0.0954 | −4.46 | – | – | – | – | – | – | −0.0436 | −3.21 |
| Bicycle Trips | | | | | 1.0000 | | −0.2189 | −7.16 | 0.1489 | 4.26 | – | – | – | – | −0.1409 | −4.18 |
| Walk Trips | | | | | | | 1.0000 | | 0.0480 | 2.14 | – | – | – | – | −0.0365 | −1.71 |
| Drive Access | | | | | | | | | 1.0000 | | – | – | – | – | 0.8181 | 181.47 |
| Transit Access | | | | | | | | | | | 1.0000 | | – | – | −0.1105 | −6.95 |
| Bicycle Access | | | | | | | | | | | | | 1.0000 | | −0.0380 | −2.47 |
| Walk Access | | | | | | | | | | | | | | | 1.0000 | |
| *Goodness of fit* | Link | | Function threshold | | Observations | | No. of dimensions | | Likelihood (mean) | | AIC | | BIC | | Function evals. | |
| | Multivariate probit | | flexible | | 5660 | | 8 | | −356723.83 | | 715207.36 | | 721050.56 | | 17,651 | |

**Table 5a**

Estimation results for household type Multi adults with one or more children- Exogenous Variable Effects.

| Variable | Auto Trips | | Transit Trips | | Bicycle Trips | | Walk Trips | | Auto Access | | Transit Access | | Bicycle Access | | Walk Access | |
|---|---|---|---|---|---|---|---|---|---|---|---|---|---|---|---|---|
| | Estimate | z value | Estimate | z value | Estimate | z value | Estimate | z value | Estimate | z value | Estimate | z value | Estimate | z value | Estimate | z value |
| *(base: Presence in age category 0–15 years)* | | | | | | | | | | | | | | | | |
| Presence in age category 16–25 years | 0.1240 | 2.15 | 0.3998 | 6.51 | – | – | 0.1186 | 1.82 | – | – | – | – | – | – | – | – |
| Presence in age category 26–35 years | – | – | – | – | −0.5448 | −3.20 | −0.1497 | −2.01 | – | – | – | – | −0.1528 | −2.71 | – | – |
| Presence in age category 36–45 years | – | – | – | – | – | – | – | – | – | – | – | – | – | – | – | – |
| Presence in age category 46–55 years | 0.2095 | 3.85 | – | – | – | – | – | – | – | – | – | – | – | – | – | – |
| Presence in age category 56–65 years | – | – | – | – | – | – | – | – | – | – | – | – | – | – | – | – |
| Presence in age category 66 and above years | – | – | −0.1773 | −2.86 | – | – | – | – | – | – | – | – | – | – | – | – |

(continued on next page)

**Table 5a** (*continued*)

| Variable | Auto Trips | | Transit Trips | | Bicycle Trips | | Walk Trips | | Auto Access | | Transit Access | | Bicycle Access | | Walk Access | |
|---|---|---|---|---|---|---|---|---|---|---|---|---|---|---|---|---|
| | Estimate | z value | Estimate | z value | Estimate | z value | Estimate | z value | Estimate | z value | Estimate | z value | Estimate | z value | Estimate | z value |
| Presence of students in the household | 0.1718 | 2.04 | 0.5612 | 5.94 | – | – | 0.5141 | 4.86 | – | – | – | – | – | – | – | – |
| Distance to CBD (km) | 0.0273 | 5.97 | −0.0193 | −3.91 | −0.0965 | −9.70 | – | – | −0.0361 | −9.03 | −0.1799 | −27.56 | −0.0938 | −19.94 | – | – |
| Population Density (Standardised value) | – | – | – | – | – | – | – | – | 0.3738 | 17.40 | 0.2148 | 5.74 | – | – | – | – |
| Employment Density (Standardised value) | – | – | −0.2868 | −2.79 | – | – | – | – | −6.3324 | −41.63 | 1.1796 | 7.64 | 0.6777 | 5.33 | −0.2968 | −11.84 |
| *(base: Income less than 15,000$)* | | | | | | | | | | | | | | | | |
| Income 15,000 $-40,000$ | – | – | 0.1772 | 1.97 | – | – | – | – | – | – | – | – | – | – | – | – |
| Income 40,000$ to 60,000$ | – | – | 0.2369 | 2.72 | – | – | – | – | – | – | – | – | – | – | – | – |
| Income 60,000$ to 100,000$ | – | – | 0.3162 | 4.01 | – | – | – | – | – | – | – | – | – | – | – | – |
| Income 100,000$ to 125,000$ | 0.3120 | 3.87 | – | – | – | – | – | – | 0.1364 | 1.81 | – | – | – | – | 0.2583 | 3.47 |
| Income greater than 125,000$ | 0.4804 | 7.89 | 0.1649 | 2.05 | – | – | – | – | – | – | – | – | – | – | – | – |
| *(base: fraction of male adults in the household)* | | | | | | | | | | | | | | | | |
| fraction of female adults in the household | – | – | 0.3776 | 2.52 | −1.0723 | −3.23 | – | – | −0.2705 | −1.79 | – | – | – | – | −0.3000 | −2.03 |
| *(base case: Vehicle availability per licensed adult is zero)* | | | | | | | | | | | | | | | | |
| Vehicle availability per licensed adult is between zero and one | 1.7255 | 12.72 | −0.8008 | −7.49 | – | – | – | – | – | – | – | – | – | – | – | – |
| Vehicle availability per licensed adults as one or more | 1.9199 | 13.89 | −1.1095 | −10.14 | – | – | −0.1334 | −2.40 | – | – | – | – | – | – | – | – |
| *(base case: Transit pass ownership less than one per adult in the household)* | | | | | | | | | | | | | | | | |
| Transit pass ownership one or more per adult in the household | −0.7694 | −5.25 | 1.0604 | 7.34 | – | – | −0.3992 | −2.65 | – | – | – | – | – | – | – | – |
| *(base case: No worker household)* | | | | | | | | | | | | | | | | |
| Single worker household | – | – | – | – | – | – | – | – | – | – | – | – | – | – | – | – |
| Multi-worker household | – | – | 0.3100 | 5.06 | – | – | – | – | – | – | – | – | – | – | – | – |
| *(base case: dwelling type house or townhouse)* | | | | | | | | | | | | | | | | |
| Apartment type dwelling | −0.2057 | −3.30 | – | – | −0.6988 | −3.79 | – | – | −0.3323 | −4.97 | −0.1693 | −2.52 | −0.3005 | −5.34 | −0.2116 | −3.32 |

**Table 5b**
Estimation results for household type Multi adults with one or more children - Endogenous Variable Effects, Correlation Effects and Goodness-of-fit.

| Variable | Auto Trips | | Transit Trips | | Bicycle Trips | | Walk Trips | | Auto Access | | Transit Access | | Bicycle Access | | Walk Access | |
|---|---|---|---|---|---|---|---|---|---|---|---|---|---|---|---|---|
| | Estimate | z value | Estimate | z value | Estimate | z value | Estimate | z value | Estimate | z value | Estimate | z value | Estimate | z value | Estimate | z value |
| *(base case: Drive Access Very Low)* | | | | | | | | | | | | | | | | |
| Auto Access Low | – | – | −0.2557 | −2.73 | – | – | – | – | NA | | NA | | NA | | NA | |
| Auto Access Medium | – | – | −0.2310 | −2.55 | – | – | – | – | NA | | NA | | NA | | NA | |
| Auto Access High | – | – | – | – | – | – | – | – | NA | | NA | | NA | | NA | |

(continued on next page)

**Table 5b** (*continued*)

| Variable | Auto Trips | | Transit Trips | | Bicycle Trips | | Walk Trips | | Auto Access | | Transit Access | | Bicycle Access | | Walk Access | |
|---|---|---|---|---|---|---|---|---|---|---|---|---|---|---|---|---|
| | Estimate | z value | Estimate | z value | Estimate | z value | Estimate | z value | Estimate | z value | Estimate | z value | Estimate | z value | Estimate | z value |
| Auto Access Very High | – | – | – | – | – | – | – | – | NA | | NA | | NA | | NA | |
| *(base case: Transit Access Very Low)* | | | | | | | | | | | | | | | | |
| Transit Access Low | – | – | – | – | – | – | – | – | NA | | NA | | NA | | NA | |
| Transit Access Medium | – | – | – | – | – | – | – | – | NA | | NA | | NA | | NA | |
| Transit Access High | – | – | – | – | – | – | 0.3658 | 4.52 | NA | | NA | | NA | | NA | |
| Transit Access Very High | – | – | – | – | – | – | 0.7669 | 7.39 | NA | | NA | | NA | | NA | |
| *(base case: Bicycle Access Very Low)* | | | | | | | | | | | | | | | | |
| Bicycle Access Low | – | – | – | – | 0.3360 | 2.74 | – | – | NA | | NA | | NA | | NA | |
| Bicycle Access Medium | – | – | – | – | – | – | – | – | NA | | NA | | NA | | NA | |
| Bicycle Access High | – | – | – | – | – | – | – | – | NA | | NA | | NA | | NA | |
| Bicycle Access Very High | – | – | – | – | – | – | – | – | NA | | NA | | NA | | NA | |
| *(base case: Walk Access Very Low)* | | | | | | | | | | | | | | | | |
| Walk Access Low | – | – | 0.2252 | 2.40 | – | – | – | – | NA | | NA | | NA | | NA | |
| Walk Access Medium | – | – | 0.2292 | 2.41 | – | – | – | – | NA | | NA | | NA | | NA | |
| Walk Access High | – | – | – | – | – | – | 0.1262 | 2.02 | NA | | NA | | NA | | NA | |
| Walk Access Very High | – | – | – | – | – | – | – | – | NA | | NA | | NA | | NA | |
| *Thresholds* | | | | | | | | | | | | | | | | |
| Intercept 1\|2 | 1.2463 | 7.86 | – | – | – | – | 0.8635 | 7.88 | −0.3671 | −3.37 | −3.6112 | −32.43 | −2.2269 | −28.56 | −0.9210 | −10.69 |
| Intercept 2\|3 | 2.0840 | 12.80 | – | – | – | – | 1.0312 | 9.38 | 0.1815 | 1.68 | −2.6058 | −25.81 | −1.5055 | −20.14 | −0.6176 | −7.12 |
| Intercept 3\|4 | 2.6300 | 16.00 | 0.7223 | 4.02 | 0.4390 | 2.16 | 1.6526 | 14.74 | 0.7624 | 7.10 | −1.4742 | −16.46 | −0.8468 | −11.83 | | |
| Intercept 4\|5 | 3.1616 | 19.13 | 1.4851 | 8.22 | 0.8014 | 3.82 | 2.3605 | 20.60 | 1.4820 | 13.62 | −0.3642 | −4.30 | | | 0.5060 | 5.83 |
| *Correlation Terms* | | | | | | | | | | | | | | | | |
| Auto Trips | 1.0000 | | −0.2584 | −10.44 | −0.1301 | −2.55 | −0.1685 | −6.01 | – | – | – | – | – | – | −0.0510 | −1.96 |
| Transit Trips | | | 1.0000 | | −0.1690 | −3.09 | −0.1580 | −5.60 | – | – | – | – | – | – | – | – |
| Bicycle Trips | | | | | 1.0000 | | – | – | – | – | – | – | – | – | – | – |
| Walk Trips | | | | | | | 1.0000 | | | | – | – | – | – | 0.0516 | 1.81 |
| Drive Access | | | | | | | | | 1.0000 | | – | – | −0.0485 | −1.82 | 0.8106 | 115.13 |
| Transit Access | | | | | | | | | | | 1.0000 | | – | – | −0.0945 | −3.55 |
| Bicycle Access | | | | | | | | | | | | | 1.0000 | | −0.0500 | −1.93 |
| Walk Access | | | | | | | | | | | | | | | 1.0000 | |
| *Goodness of fit* | Link | | Function threshold | | Observations | | No. of dimensions | | Likelihood (mean) | | AIC | | BIC | | Function evals. | |
| | Multivariate probit | | flexible | | 2138 | | 8 | | −144319.97 | | 290019.88 | | 293930.37 | | 19,144 | |

## References


Agyemang-Duah, K., Hall, F.L., 1997. Spatial transferability of an ordered response model of trip generation. Transp. Res. A 31 (5), 389–402.

Agyemang-Duah, K., Hall, F.L., Anderson, W.P., 1995. Trip generation for shopping travel. Transp. Res. Rec. 1493, 12–20.

Aitchison, J., Silvey, S.D., 1957. The generalization of probit analysis to the case of multiple responses. Biometrika 44 (1/2), 131–140.

Allen, J., Farber, S., 2020. Planning transport for social inclusion: An accessibility-activity participation approach. Transp. Res. Part D: Transp. Environ. 78, 102212.

Bagley, M.N., Mokhtarian, P.L., 2002. The impact of residential neighbourhood type on travel behaviour: A structural equations modelling approach. Ann. Reg. Sci. 36 (2), 279–297.

Bhat, C.R., Guo, J.Y., 2007. A comprehensive analysis of built environment characteristics on household residential choice and auto ownership levels. Transp. Res. B 41 (5), 506–526.

Bhat, C.R., Astroza, S., Bhat, A.C., Nagel, K., 2016. Incorporating a Multiple Discrete-Continuous Outcome in the Generalized Heterogeneous Data Model: Application to Residential Self-Selection Effects Analysis in an Activity Time-use Behaviour Model. Transp. Res. B 91, 52–76.

Bhat, C.R., Guo, J., 2004. A mixed spatially correlated logit model: formulation and application to residential choice modeling. Transp. Res. B Methodol. 38 (2), 147–168.

Boarnet, M.G., Bostic, R.W., Rodnyansky, S., Burinskiy, E., Eisenlohr, A., Jamme, H.T., Santiago-Bartolomei, R., 2020. Do high income households reduce driving more when living near rail transit? Transp. Res. Part D: Transp. Environ. 80, 102244.

Boisjoly, G., El-Geneidy, A.M., 2017. How to get there? A critical assessment of accessibility objectives and indicators in metropolitan transportation plans. Transp. Policy 55, 38–50.

Brown, A.E., Blumenberg, E., Taylor, B.D., Ralph, K., Voulgaris, C.T., 2016. A taste for transit? Analyzing public transit use trends among youth. J. Public Transp. 19 (1), 49–67.
